# A detailed review of pharmacokinetics/pharmacodynamics of progestogens in oral contraception

**DOI:** 10.3389/fendo.2026.1730768

**Published:** 2026-03-26

**Authors:** Ulysse Gaspard, Guillaume Chatel, Jonathan Douxfils, Frank Z. Stanczyk

**Affiliations:** 1 Department of Obstetrics and Gynecology, University of Liege, Liege, Belgium; 2 Estetra SRL, a member of Gedeon Richter PLC, Liege, Belgium; 3 Research Unit in Clinical Pharmacology and Toxicology (URPC), NAmur Research Institute for LIfe Sciences (NARILIS), Faculty of Medicine, University of Namur, Namur, Belgium; 4 QUALIresearch, Qualiblood s.a, Liège, Belgium; 5 Department of Biological Hematology, Centre Hospitalier Universitaire Clermont-Ferrand, Hôpital Estaing, Clermont-Ferrand, France; 6 Department of Obstetrics and Gynecology, University of Southern California, Los Angeles, CA, United States

**Keywords:** hemostasis, oral contraception, pharmacodynamics, pharmacokinetics, progestogens, thromboembolism

## Abstract

Many different progestogens are used by millions of women worldwide for oral contraception, either alone (Progestogen-only Pills [POPs]) or as combined oral contraceptives (COCs) comprising a synthetic estrogen (ethinyl estradiol [EE]), or a natural estrogen (estradiol and its valerate [E2; E2V] or estetrol [E4]), associated with a progestogen. This review describes first the three families of progestogens derived either from testosterone, 17 alpha-hydroxyprogesterone or spironolactone. Their pharmacokinetic parameters are largely differing, but also their metabolism, potency and efficacy via many steroid receptors, thereby confirming the absence of a class effect of these progestogens. In the pharmacodynamic section, POPs will be described in detail, showing high efficacy and safety, though low cyclic tolerance. When different progestogens are combined with EE in COCs, and despite high efficacy, tolerability and improved cyclic tolerance compared with POPs, safety is hampered, among other by vascular thromboembolism risks (venous [VTE] as well as arterial [ATE]). These will be analyzed with the help of most recent results establishing that EE/levonorgestrel (LNG) entails less adverse vascular events than other EE-containing COCs. Also, in the last 15 years, COCs containing natural estrogens (E2V/dienogest (DNG) and E2/nomegestrol acetate (NOMAC)) have shown through meta-analyses of clinical thrombotic events and adequate hemostatic studies, a lower VTE risk than with use of EE/LNG. Moreover, another natural estrogen-containing COC, E4/drospirenone (DRSP), predicts also a low level of risk through global hemostasis assessments, disproportionality analyses and other studies. So, a new possibility arises that the safest COCs in terms of thrombotic risk might be the natural estrogen-containing COCs, where the estrogen is combined to one of three different non-androgenic progestogens.

## 1 General introduction

Originally, progestogens comprising progesterone (P4) and synthetic progestogens, also called progestins, were investigated as compounds to maintain pregnancy: they were designated as pro-gestational hormones, which is the origin of the name progestogen. Development of numerous progestogens in the 1960s showed no evidence for progestogens to support pregnancy, while only P4 had this capacity. Indeed, endogenous P4 shows essential functions not only for maintaining pregnancy, but also acts on the genital organs, central nervous system, pituitary and the breast, among others. However, P4 administered orally is rapidly metabolized, mainly in the intestinal tract and liver, leading to lower bioavailability, with ensuing minimal effects (1). Accordingly, synthetic progestogens differ from P4 mainly in view of an oral administration and a better bioavailability, and were developed with essentially marked progestogenic effects, allowing them to be used in rather low dosages with a concomitant reduction in main side effects. These numerous progestogens, in addition to their binding to the progesterone receptor (PR), may bind to other receptors, display very different properties than P4 and lack a progestogen class effect: as Stanczyk stated “All progestogens are not created equal” (2, 3).

Although progestogens can be used as therapy, a frequent use of progestogens is hormonal contraception: combination of an estrogen and a progestin strongly inhibits gonadotropin secretion and ovarian function, leading to reversible fertility prevention. This combined estrogen-progestin contraception can be not only oral (combined oral contraceptive, COC), but also transdermal, transvaginal, or intramuscular. Moreover, progestogen-only contraception, i.e., without combined estrogen, whether oral (progestogen-only pill, POP), subdermal (implants) or intrauterine (intrauterine devices, IUDs) are available. POPs can be equally effective as COCs, because progestins administered alone, even low-dose, may suppress ovulation fully or partly. They also alter cervical mucus, endometrial receptivity and tubal motility, thus enhancing contraceptive effectiveness (4). Additionally, some progestins through specific properties such as anti-androgenic effects are useful in treating hyperandrogenism (e.g., polycystic ovarian syndrome), or through anti-mineralocorticoid action to alleviate, for instance, premenstrual dysphoric disease and blood pressure disorders (5, 6). Further to contraception itself, COCs and POPs display probably more than twenty subjective or objective non-contraceptive health benefits (7).

The present chapter is divided into two sections. In the first section, we will review some historical aspects of the development of progestins and summarize key points of their structure-function relationships and mode of action, whether being alone or combined with an estrogen. We will then envisage specific pharmacokinetic (PK) parameters of these progestins to analyze the pharmacologic effect they entail as POPs or COCs (8). Subsequently, the second section will deal with pharmacodynamics (PD) of progestogens, after a critique of the still used “generation concept” for classification of progestogens (9). The clinical impact and selected important adverse effects and risks of COCs and POPs (notably on hemostasis) will be addressed, in addition to a description of specific characteristics of each progestin involved in oral hormonal contraception.

## 2 Key historical and pharmacological aspects of the development of progestogens

The different progestogens available on the market differ by several pharmacological aspects which are associated with their chemical structure (**Table 1**). The development of the different chemical entities in the field of progestogens started with the extraction of natural estrogens and P4, which were first extracted in minute amounts from thousands of sow ovaries and their injection to a variety of animals led to the inhibition of fertility. Their chemical structure was progressively identified, and estrogen compounds were already developed since the 1920s: apart from estradiol (E2) and estrone (E1), potent synthetic derivatives like diethylstilbestrol (DES), and ethinyl estradiol (EE) were developed later, and used in high doses with concomitant adverse effects.

**Table 1:** Classification of progestogens according to molecular structure.

Origin	Progestogen Classification				
Natural	Progesterone				
Synthetic	Structurally related to	Category	Progestogen	Abbreviation	Alone or combined with the following estrogens in oral contraceptives
	Testosterone	Ethinylated derivatives, estranes	Norethisterone (Norethindrone), Norethisterone acetate	NET, NETA	Alone or with EE
		Ethinylated derivatives, 13-ethyl-gonanes	Levonorgestrel	LNG	Alone^*^ or with EE
			Norgestimate	NGM	EE
			Desogestrel	DSG	Alone or with EE
			Gestodene	GSD	EE
		19-nortestosterone non-ethinylated derivative	Dienogest	DNG	Alone or with EE or with E2V
	Progesterone	Pregnane derivatives, acetylated	Chlormadinone acetate	CMA	EE
			Cyproterone acetate	CPA	EE
		19-norpregnane derivative, acetylated	Nomegestrol acetate	NOMAC	E2
	Spironolactone		Drospirenone	DRSP	Alone or with EE or E4

^*^ Recently, norgestrel, a racemic mixture of D-(-)-norgestrel (levonorgestrel, a potent progestogen), and L-(+)-norgestrel (dextronorgestrel, hormonally inactive), was introduced as a POP in USA.

In the case of P4, in 1943, Russell Marker at Syntex succeeded in preparing this steroid from diosgenin, a plant steroid easily extracted from yams. Later, industrial synthesis of P4 was obtained from E1 (10). However, oral administration of P4 was followed by rapid inactivation during the first hepatic pass, since P4 had to be injected to preserve its hormonal activity. The need for orally active progestogens resulted in developing synthetic products which demonstrated to be rather well tolerated.

In an attempt to develop an orally potent androgen, the Inhoffen and Hohlweg (Schering) developed 17-alpha ethinyl testosterone (ethisterone) which surprisingly showed not only an attenuated androgenic activity but also an unwanted considerable progestogenic action (11).

### 2.1 Development of progestogens structurally related to testosterone

When applying the Birch reaction to ethisterone, Carl Djerassi (Syntex) in 1951 obtained 19-nor-ethinyltestosterone, i.e., norethisterone (or norethindrone) (NET), which turned out to be a highly active, well-tolerated progestogen (20 times more active than ethisterone) with a low androgenic activity (12, 13). In the same year, Frank Colton (Searle) developed norethynodrel (NED), a 19-nortestosterone estrane, which was the first active progestational agent to receive a patent. NED is a prodrug, which is rapidly converted to NET. Under the input of Gregory Pincus in the mid-1950s, the first human trials of NED for contraception were conducted in 1956 in Puerto Rico (by Garcia and Rice-Wray). However, purified NED alone yielded breakthrough bleedings, and it was decided to add an estrogen (3-methylether of EE, i.e., mestranol-which is readily converted to EE after absorption). Thus, the principle of combined estrogen-progestogen oral contraception was established. Accordingly, this combination, called Enovid^®^ was approved in the US and marketed by Searle first for miscarriages and menstrual disorders and later allowed for contraception in 1960. Enovid^®^ was high dose: 150 µg mestranol/9.85 mg NED. Ortho-Novum^®^ containing NET/EE (from Syntex) became available in 1962. In the 1960s, lynestrenol and ethynodiol diacetate, were developed as NET prodrugs.

In 1968, Wyeth Laboratories synthesized norgestrel (NG), a racemate mixture of dextronorgestrel (an inactive enantiomer), and levonorgestrel (LNG), the active enantiomer usually used in combination with EE, or alone, as a POP. The important characteristic of NG relies on the replacement of the methyl group at carbon-13 of NET by an ethyl group. In so doing, this 19-nortestosterone ethinylated compound becomes a 13-ethyl-gonane with potent progestogenic and also androgenic properties.

In the 1970s, the family of 13-ethyl-gonanes was extended by the development of desogestrel (DSG), a prodrug of etonogestrel (ENG). Similarly, gestodene (GSD) developed by Schering, is an active 13-ethyl-gonane and not a prodrug of LNG.

Dienogest (DNG), a 19-nortestosterone derivative (but not a 13-ethylgonane), was developed by Hubner and Ponsold for Jena Pharm (Germany) in 1978. It is the only progestogen with replacement of the C-17 alpha ethinyl group by a cyanomethyl group.

Later on, in the 1980s, norgestimate (NGM), another 13-ethyl gonane was developed as a prodrug, which is rapidly converted after oral administration to LNG-3-oxime (norelgestromin, NGMN), LNG-17 beta acetate, as well as LNG.

### 2.2 Development of progestogens structurally related to progesterone

In 1954, Karl Junkmann, at Schering, developed the first progesterone derivative by carbon 17-acetylation, which partly preserved some progestational activity. In the following years, addition of a methyl group at C6 to this acetoxyprogesterone allowed the development of compounds with substantial oral progestogenic activity (megestrol acetate, MGA) at Syntex, in 1959. MGA was developed together with chlormadinone acetate (CMA). Both steroids were potent progestogens, with activity very near to cyproterone acetate (CPA), which was developed by R. Wiechert at Schering in 1961. CPA differs from CMA only by a methylene group attached to C1–2 and has also the most potent anti-androgenic activity compared to all other progestogens. MGA, CMA, and CPA altogether form the pregnane group. Moreover, medroxyprogesterone acetate (MPA), also a pregnane, is not used as a COC or POP, but more specifically as an intramuscular/subdermal progestogen-only for contraception.

In 1944, Max Ehrenstein had succeeded in removing the methyl group on carbon 10 from P4, obtaining a 19-norprogesterone with potent progestogenic effects when administered non-orally (14). This inaugurated the 19-nor-pregnane family, which was developed later in the 1970s and the 1980s with nomegestrol acetate (NOMAC). NOMAC is now used in an oral COC, in association with E2 instead of EE. Other 19-nor-pregnanes were developed thereafter, including demegestone, promegestone, and trimegestone, all potent oral progestogens, while segesterone acetate (SGT) was not orally active. SGT is also a potent progestogen combined with EE for vaginal administration as a female contraceptive, whereas in men, SGT combined with testosterone in a gel was developed as a male contraceptive.

### 2.3 Development of a progestogen structurally related to spironolactone

Drospirenone (DRSP) is derived from spironolactone and is structurally close to spironolactone. It was synthesized by R. Wiechert in 1976 at Schering, though its pharmacological potential was only discovered 20 years later. It is an orally potent progestogen with distinct, strong anti-mineralocorticoid activity, and rather weak anti-androgenic activity (15). It was marketed in 2000 combined with EE and recently combined with the native estrogen estetrol (E4), since 2021. Moreover, DRSP alone is used as a POP since 2019 (16, 17).

Indeed, this remarkable saga, which practically began in the 1950s, was developed by daring biochemists and open-minded clinicians. It opened the way for women desiring to master their fertility by themselves. It is up to us, scientists and physicians, to persistently make progress toward more efficacy, safety and tolerability of contraception.

## 3 Pharmacokinetic characteristics of progestogens for oral contraception either alone or in combination with estrogens

### 3.1 Classification and structure of the selected progestogens

The first progestogens essentially used for contraception were derivatives of either testosterone or 17-hydroxyprogesterone. Their structure was often associated with androgenic or corticoid-like effects and other effects due to interactions not only with PR, but also with estrogenic, androgenic, glucocorticoid or mineralocorticoid receptors (ER, AR, GR, MR), whether as agonist or antagonist agents. Later, new progestogens were developed with, notably, less metabolic and androgenic impact, increasing tolerability (2).

**Figure 1:**
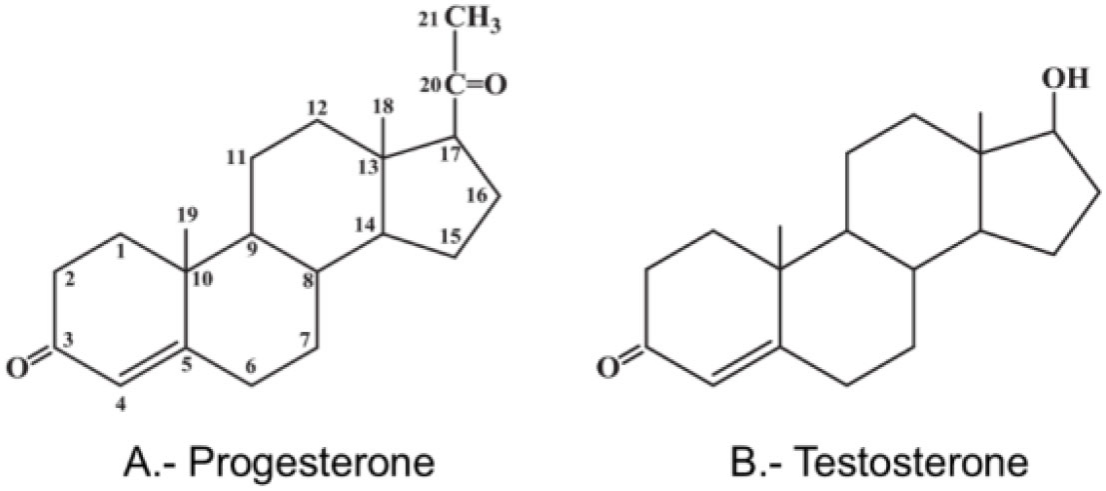
Structural formulae of progesterone and testosterone.

**Figure 2:**
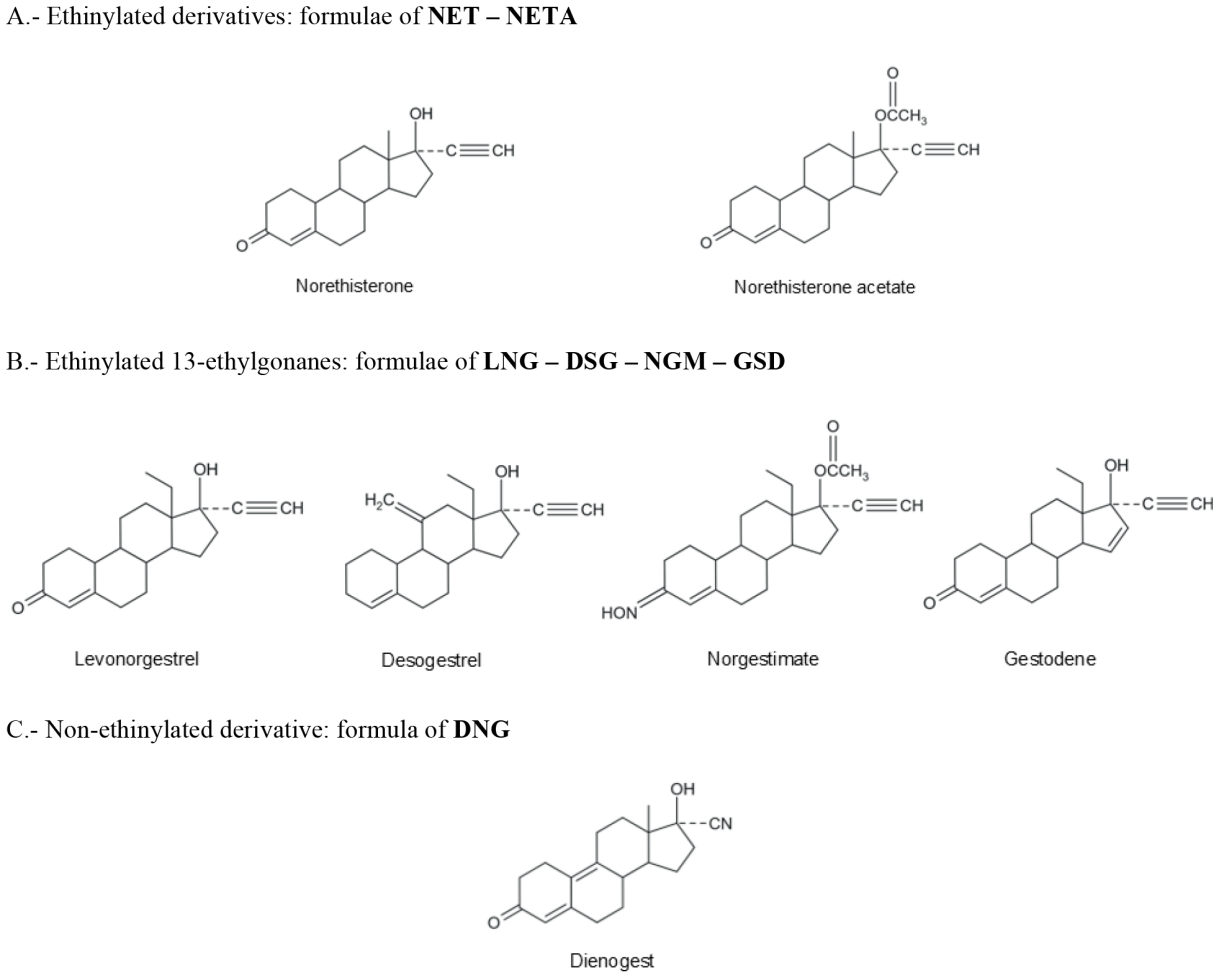
Formulae of progestogens structurally related to testosterone used in oral contraception.

**Figure 3:**
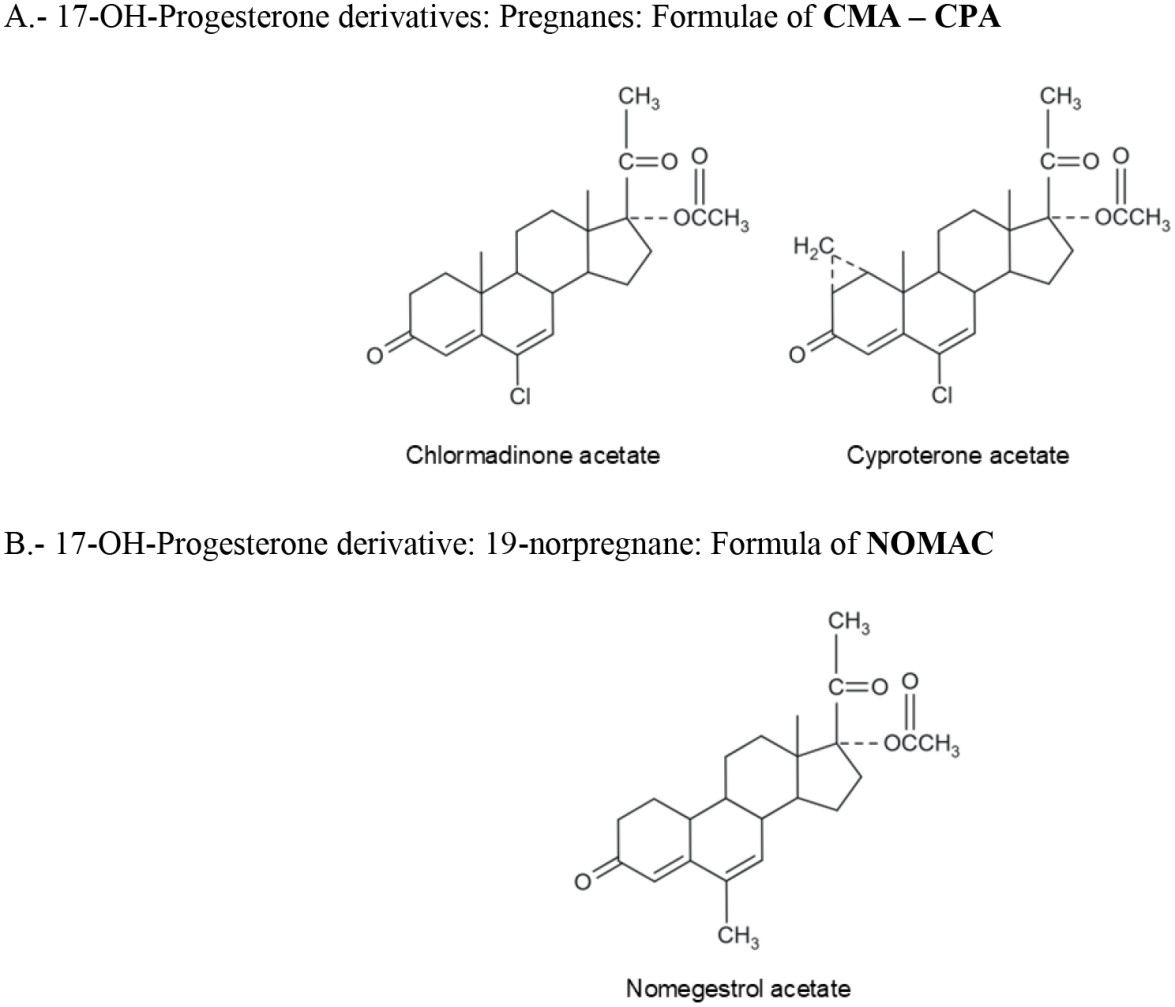
Formulae of progestogens structurally related to 17-hydroxyprogesterone used in oral contraception.

**Figure 4:**
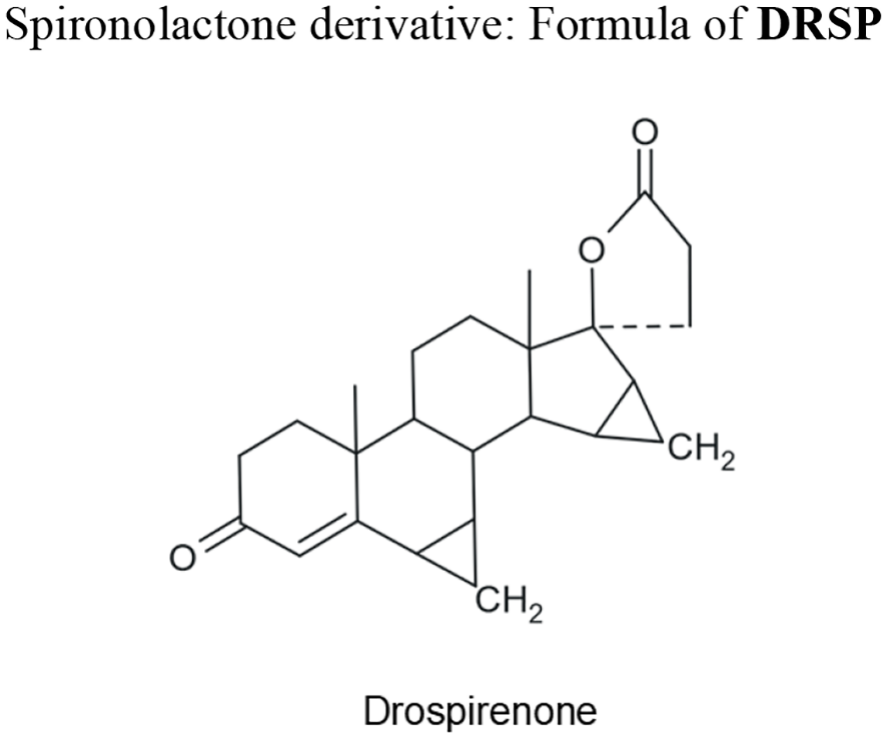
Formula of progestogen structurally related to spironolactone used for contraception.

It is necessary, for reasons of clarity, to classify the progestogens that we use nowadays for oral contraception (**Table 1**) and to show their structural formulae to better delineate their PK characteristics and PD function, as shown later in this chapter (**Figures 1**–**4**).

As shown in **Table 1**, progestogens used in contraception can be divided into 2 general types: natural and synthetic (progestins); their chemical structures are shown in **Figures 1**–**4**. There is only one natural progestogen and that is P4 (**Figure 1**). The progestins can be subdivided into those structurally related to P4, those related to testosterone, and one that is related to spironolactone (**Table 1**). The structure of testosterone is shown in **Figure 1**. This does not mean that they are synthesized from those 2 steroids, but only that they are related in chemical structure. Those related to P4 have 20 or 21 carbons in their base structure and those related to testosterone have 18 or 19 carbons.

In contrast to the progestins structurally related to P4 (**Figures 3A, B**), those related to testosterone can be divided into those that contain an ethinyl group and those that do not (**Figures 2A–C**). Progestins containing an ethinyl group can be subdivided into those that have an ethyl group on carbon 13 (called 13-ethylgonanes or levonorgestrel family) and those that retain the methyl group (called estranes or norethisterone family). The NET family of progestins consists of inactive NET derivatives that are rapidly converted to NET during first hepatic pass metabolism after oral intake; they include NED, norethisterone acetate (NETA), ethynodiol diacetate and lynestrenol. Inactive compounds that are converted to active compounds are referred to as prodrugs. In contrast to the NET family of progestins, the levonorgestrel family of progestins contains 3 compounds, namely DSG, NGM, and GSD; the first 2 are prodrugs. DSG is converted to its active form, ETN (formerly called 3-ketodesogestrel), and NGM is converted to 2 active forms, namely NGMN (formerly called levonorgestrel-3-oxime) and LNG. There is one non-ethinylated progestin that is structurally related to testosterone, and that is DNG (**Figure 2C**), which contains a cyanomethyl group instead of an ethinyl group at carbon 17.

Progestogens related in chemical structure to P4 can be classified into pregnane and 19-norpregnane categories (**Figures 3A, B**). The pregnane category includes progestins that have a basic steroid nucleus similar to P4 (case of CMA and CPA), whereas the norpregnanes lack a methyl group at carbon 10 (case of NOMAC). Each subcategory can be subdivided further into those progestins that have an acetate group and those that do not possess this group. In a third category of progestins, there is DRSP, which is a spironolactone derivative (**Figure 4**).

In this review, COCs are not classified on a “generation” basis for the following reason: Early in the 1990s, pharmaceutical companies introduced a classification of COCs based on a “generation concept” implying that the newer compounds were safer or “better”. These well-known generations showed a trend (1) to progressively decrease the EE content of COCs and potentially decreasing the thrombotic risk, and (2) to reduce androgenicity of progestogens for a better tolerability, the fourth generation even containing an anti-androgenic progestogen (DRSP). However, this classification is incomplete and does not comprise COCs containing CMA, CPA, or DNG, nor COCs containing estrogens other than EE (i.e., E2, estradiol valerate (E2V), or E4). Moreover, this classification – established only on the time of introduction of these COCs to the market – does not include a gradation according to biological, metabolic, hemostatic, or epidemiological impact of COCs.

This led to a high degree of controversy, such as the one reported by Creinin and Jensen: The use of “generations” to define COCs is not only obsolete and largely incomplete but is a “marketing idea” that has confused clinicians for years, because it does not provide a valid differentiation of product safety or efficacy (9). It would be more accurate if clinicians refer to the various estrogens and progestogens according to the usual nomenclature (e.g., estrane, 13-ethylgonane, etc.) and also follow clinical trials for evaluating the combined products. In our view, it is not impossible that in the near future, a classification of all COCs could be established in great part according to their thrombotic risk – which is a major problem. This seems possible to be reached through an adapted and validated hemostasis global test (18).

### 3.2 Basal characteristics of pharmacokinetics of progestogens and their affinities for steroid receptors

Investigating the PK of active substances during drug development is necessary to better understand the biological action of the compounds and even anticipate their behavior under certain conditions. The PK of progestogens given orally are influenced by the route of administration, bioavailability, metabolism, binding to serum proteins (ADME or Absorption, Distribution, Metabolism, and Excretion). All of them impact the concentration of drug over time. When measuring the concentration of a drug, several parameters are typically calculated such as time (Tmax) of the highest concentration (Cmax), the total amount of drug available in the bloodstream as a function of time (AUC: area under the curve), and terminal half-life (T_1/2_) (reported in **Table 2**; **Supplementary Table**). These PK parameters are derived from measurements of progestins in serum or plasma samples obtained at frequent intervals from women during 24 h or longer after oral dosing of a progestogen. However, there is a limited number of valid studies on the PK of progestins due to deficiencies in such studies. There are deficiencies in study design, sample size, and assay methodology. PK parameters may be influenced by extrinsic (e.g., drug interactions, disease, etc.) and intrinsic (e.g., genetics, food, etc.) factors (8).

**Table 2:** PK characteristics of progestogens.

Progestogens	Oral dose (mg)	Bioavailability (%)	Half-life (h)	Albumin-bound (%)	SHBG-bound (%)	CBG-bound (%)	References
P4	100 200	<5	16.2-18.3	79.3	0	17.7	(1, 2, 19, 20)
NET	1	64	8-10	60.8	35.5	0	(21–24)
LNG	0.150 0.250	87-100	11.9-37.6	50.0	47.5	0	(22, 25–33)
NGMN (active form of NGM)	0.250	ND	15.6-24.9	97.2	0	0	(34–37)
ENG (active form of DSG)	0.150	62-82	11.9-30.6	65.9-69.2	29.0-31.6	0	(38–43)
GSD	0.060 0.075	87.0-99.3	12-22	11.0-24.1	75.3-88.2	0	(40–42, 44–47)
DNG	2 3	90.5-96.2	10.8-11.6	91	0	0	(48)
CMA	2	100	25.3-39.1	97-99	0	0	(49–52)
CPA	2	88-100	40.8-78.6	92-93	0	0	(10, 53–55)
NOMAC	2.5	63	36.2-50	97.5-98	0	0	(56–58)
DRSP	3 4	76	30	95-97	0	0	(59–61)

Oral administration of progestogens may be associated with an important first hepatic pass effect, which is the transformation of the drug to metabolites following contact with the intestinal bacteria and mucosa and liver before reaching the circulation. It leads to a reduced concentration of the active drug compared with the parenteral administration of the same drug. The importance of the first hepatic pass effect varies from one progestogen to another, which yields a high bioavailability close to 100% for some progestogens (e.g., LNG, CMA, CPA, etc.) while others are lower ranging from 63 to 76% (e.g., NET, NOMAC, DRSP, etc.) (**Table 2**).

Once in the circulation the administered drug may form complexes with plasma proteins, often considered as a possible reservoir. Progestogens tend to form complexes with albumin, sex hormone binding globulin (SHBG), and/or cortisol binding globulin (CBG). Binding to SHBG or CBG with high affinity is limiting accessibility of the drug to target tissues and metabolism. In contrast, the binding to albumin is with low affinity, which does not impact the interaction of progestogens with metabolic enzymes and receptors at the site of action (**Table 2**).

Another informative parameter collected from a drug is its half-life, which refers to the time taken for the concentration of the drug to decrease by 50%. It is an indicator of the time the biological action is expected to remain, which is important for oral contraceptives in case of a missed pill or vomiting.

As COCs, progestogens are combined with either EE or natural estrogens, therefore their PK is influenced not only by their specific bioavailability, binding to plasma proteins, affinity for diverse receptors, but also by the estrogen. The **Supplementary Table** displays concentrations of Cmax, Tmax, and AUC of the progestogens combined with estrogens after multiple dose administration, which helps to understand the clinical aspects of COCs (62).

The diverse structures of progestogens not only allow them to have different affinities for different transport proteins in the plasma, but also, in a non-surprising way, these progestogens cross-react with several members of the steroid receptor family, because the PR, AR, GR, MR and ER, all share significant structural homology. Accordingly, the relative binding affinity (RBA) of progestogens with these receptors is greatly variable and justifies their biological effects which will be discussed in more detail in the pharmacodynamic part of this chapter. **Table 3** shows this variability of the RBA, which also depends on different methods of determination (different cell lines, animal or human tissue models), rendering this determination rather approximate, although supporting “several valuable insights” (1). In addition, **Table 4** depicts the patterns of hormonal activities and their effectiveness for P4 and synthetic progestogens, a pattern that largely correspond to their RBA.

**Table 3:** Relative binding affinities of progestogens to steroid receptors. Adapted from (10, 17).

Progestogen	PR	AR	ER	GR	MR
NET	75	15	0	0	0
LNG	150	45	0	1	75
NGMN	8^*^	-^**^	-^**^	-^**^	-^**^
ETN	150	20	0	14	0
GSD	90	85	0	27	290
DNG	5	10	0	1	0
CMA	67	5	0	8	0
CPA	90	6	0	6	8
NOMAC	125	19^***^	0	6	0
DRSP	35	65	0	6	230

PR, progesterone receptor; AR, androgen receptor; ER, estrogen receptor; GR, glucocorticoid receptor; MR, mineralocorticoid receptor

^*^(63)

^**^Data not available

^***^(64)

**Table 4:** Patterns of hormonal activities of natural progesterone and synthetic progestogens. Adapted from (1, 10, 59).

Progestogen	Progesto- genic	Anti-gonado- tropic	Anti- estrogenic	Estrogenic	Androgenic	Anti- androgenic	Gluco- corticoid	Anti-mineralo- corticoid
Natural progestogen								
Progesterone	+^*^	+	+	–	–	(+)	+	+
19-nortestosterone derivatives								
Norethisterone	+	+	+	+	+	–	–	–
Levonorgestrel	+	+	+	–	+	–	–	–
Norgestimate	+	+	+	–	+	–	–	–
Etonogestrel	+	+	+	–	+	–	–	–
Gestodene	+	+	+	–	+	–	+	+
Dienogest	+	+	+	–	–	+	–	–
17-OH-progesterone Pregnane derivatives								
Chlormadinone Ac^**^	+	+	+	–	–	+	+	–
Cyproterone Ac	+	+	+	–	–	++	+	–
19-norprogesterone Norpregnane derivative								
Nomegestrol Ac	+	+	+	–	–	(+)	–	–
Spironolactone derivative								
Drospirenone	+	+	+	–	–	+	–	+

^*^+, effective; (+), weakly effective; –, not effective.

^**^Ac, Acetate.

Following their first pass effect and circulation into the body, the progestogens are metabolized. Metabolism participates in the transformation and elimination of the drug from the body. For most drugs, metabolism can be divided between Phase I (oxidation, reduction, hydroxylation, hydrolysis) and Phase II conjugation. The Phase I enzymes generate metabolites than can retain activity and present substrates to conjugating enzymes (65). Progestogens typically undergo reduction through NADPH-dependent oxidoreductase and aldo-keto reductase family enzymes as well as oxidation by CYP enzymes (66). The Phase II enzymes generally lead to drug inactivation and render the drug more soluble, facilitating its elimination in urine/feces. The metabolism of progestogens has not always been extensively described, however, in general it follows the classical Phase I and II principles.

### 3.3 The PK characteristics of progestogens

NET is a potent progestogen with moderate androgenic activity. However, NET has no glucocorticoid or mineralocorticoid activities (see **Table 4**) (10). NETA is a prodrug, rapidly deacetylated to form NET in the intestinal tract and liver following oral administration (49). The bioavailability of NET is around 64% due to first pass metabolism (**Table 2**). NET is extensively metabolized through phase I and II enzymes. The main step consists in reduction leading to formation of dihydro and tetrahydro metabolites. Sulfate and glucuronide conjugation participate in the elimination of this drug, with sulfate metabolites being mainly present in the serum while the glucuronide metabolites represent majority of the transformed drug found in urine (66). NET is also converted to active estrogen, EE. In one study this conversion showed an equivalent dose of about 4-6 μg EE per 1 mg NET/NETA administered orally (67). Another study shows a conversion ratio of NETA to EE ranging from 0.20 to 0.33% (68). Although this conversion value yields subclinical levels of EE from low doses of NETA (1–2 mg/day), use of higher doses of NETA (10–20 mg/day) administered chronically may equate to the 20-30 µg EE range, which is clearly a concern (68). In blood, NET is bound to albumin (60.8%) and SHBG (35.5%). The half-life of NET is short compared with other progestogens (8-10h) (**Table 2**). It is noteworthy that other 19-nortestosterone ethinylated estranes such as lynestrenol, ethynodiol diacetate, and NED are also prodrugs of the active progestogen NET.

LNG. LNG is part of the racemate D,L-norgestrel (**NG**), which consists in equal parts of the potent progestogen LNG and the hormonally inactive dextronorgestrel. Therefore 0.5 mg NG is identical to 0.25 mg LNG, a progestogen with androgenic and anti-estrogenic properties, but no glucocorticoid or anti-mineralocorticoid effects (**Table 4**). LNG is not converted to EE or any other estrogen. It is not subject to first hepatic pass metabolism when administered orally, with a bioavailability ranging from 87% to 100% (**Table 2**). LNG is reduced to form dihydro and tetrahydro metabolites (69). Glucuronidated LNG metabolites are present predominantly in urine and sulfated and untransformed LNG are found in serum (66). In blood, LNG is bound to albumin (50%) and SHBG (47.5%). The half-life of LNG is very variable and can range from 11.9h to 37.6h when given orally combined with EE (**Table 2**). Some studies showed that the LNG half-life significantly increases in obese women (25, 70).

NGM is a complex prodrug that is converted into LNG-17-acetate and NGMN (norelgestromin, which is also known as levonorgestrel-3-oxime or 17-deacetylnorgestimate) (66). LNG-17-acetate and NGMN can be converted into LNG. Comparing subjects treated with NGM and LNG, it was estimated that about 22% of the dose of NGM administered became systemically available as LNG (71), meaning that LNG contributes to the pharmacological action in addition to NGMN. NGM shows lower affinity for the PR than LNG and it is also the case for NGMN and LNG-17 acetate (10). The information on NGMN is more limited; nevertheless, it has been shown to be a substrate of CYP enzymes (72). Contrary to LNG, NGMN does not bind to SHBG, and approximately 97.2% is bound to albumin (**Table 2**). The half-life of NGMN ranges from 15.6h to 24.9h in subjects treated with NGM combined with EE (**Table 2**). NGM is used as an oral estrogen-progestogen combination of EE 35 µg/NGM 250 µg, while NGMN is used in a transdermal patch combined with EE (203 µg and 34 µg µg of daily release, respectively).

DSG, which is a 13-ethyl gonane, is also a prodrug, that is converted to ENG (also called 3-keto-desogestrel), which is pharmacologically active. Note that the prerequisite for the progestogenic activity of a steroid is the existence of a 3-keto group and a double bond between C4 and C5 in the A-ring (Δ^4^-3-keto group). The 19-nortestosterone derivatives which lack this characteristic (NED, lynestrenol, DSG, and NGM) are all prodrugs, rapidly converted after oral administration to active progestins with a Δ^4^-3-keto group (10, 73). In addition to its progestogenic activity, ENG has weak androgenic activity, binds to the GR with about 14% of the affinity of dexamethasone and has weak glucocorticoid activity (19). The oral bioavailability of ENG ranges from 62% to 82% (**Table 2**). ENG is metabolized by CYP enzymes (74, 75). It is mainly bound to albumin (65.9-69.2%) but also to SHBG (29.0-31.6%). Following oral administration of DSG combined with EE, the half-life of ENG ranges from 11.9h to 30.6h (**Table 2**).

GSD is an active 13-ethyl gonane progestogen structurally related to LNG. It differs from LNG only by the presence of a double bond between C15 and C16. GSD is not subject to the first pass effect when administered orally, with a bioavailability ranging from 87 to 99.3% (**Table 2**). GSD is reduced to form dihydro and tetrahydro metabolites (66). It has additionally mild anti-mineralocorticoid and anti-estrogenic activities. GSD is highly bound to SHBG (75.3-88.2%) and to a lower extent to albumin (11.0-24.1%). After oral administration of GSD/EE, the half-life of GSD ranges from 12h to 22h (**Table 2**) (76, 77).

DNG is a 19-nortestosterone non-ethinylated progestogen characterized by a cyanomethyl group (-CH2CN) instead of an ethinyl group at C17. DNG is the only 19-nortestosterone derivative which exerts no androgenic action, but has a marked anti-androgenic activity, in addition to progestogenic properties (10). The bioavailability of DNG is high (more than 90%) (**Table 2**). DNG is metabolized by CYP3A4 leading to the formation of hydroxylated DNG, the major identified metabolite being 11ß-OH-DNG (48). Glucuronidated and sulfated conjugated forms of DNG were also identified in urine samples from women administered with DNG (66). In blood, DNG is bound to albumin (91%) and not to SHBG, leaving 9% unbound drug. When combined with EE or E2V, the half-life of DNG ranges from 10.8h to 11.6h (**Table 2**).

CMA is an active progestogen derived from 17-hydroxyprogesterone, a pregnane, with marked progestogenic effects and also anti-androgenic properties, equivalent to about 20% of the anti-androgenic effect of CPA, the most potent anti-androgenic progestogen (15). CMA also has low glucocorticoid and no mineralocorticoid activities (**Table 4**) (2, 15). Similar to other progestogens, CMA accumulates in fat tissue, genital organs, etc., with a relatively low clearance, leading to a 74% excretion in the 7 days following its administration (78). CMA is not subject to a first pass effect and exerts bioavailability of around 100% (**Table 2**). CMA is subjected to Phase I and II metabolism with formations of 14 metabolites in urine, bile and feces. The main metabolites in human plasma are hydroxylated metabolites (66). CMA is bound to albumin (97-99%) and not SHBG. The half-life of CMA ranges from 25.3h to 39.1h when administered orally in combination with EE (**Table 2**).

CPA is an active progestogen derived from 17-hydroxyprogesterone (pregnane) and has the highest anti-androgenic activity due to a competitive inhibition of the binding of endogenous androgens to the AR receptor, a dose-dependent effect of CPA. It may have some glucocorticoid activity not clearly demonstrated (10, 15). Its accumulation in fat tissue leads to a depot effect which is also dose-dependent (10). CPA is not subjected to first pass metabolism and exerts a high bioavailability of 88-100% (**Table 2**). In terms of metabolism, CPA is hydroxylated and acetylated, 15β-hydroxy-CPA being the major metabolite, which retains progestogenic activity (66). CPA is bound to albumin (92-93%) and not to SHBG. The half-life of CPA is long, ranging from 40.8h to 78.6h when administered orally in combination with EE (**Table 2**).

NOMAC is an active progestogen derived from 17-hydroxyprogesterone, which differs from MGA only by the lack of the angular C19-methyl group. It is accordingly a 19-norpregnane. It shows a progestogenic activity and also an anti-androgenic effect, which represents around 25-30% of the CPA anti-androgenic action (15). The bioavailability of NOMAC is around 63% (**Table 2**). NOMAC is metabolized through hydroxylation dependent on CYP3A3, CYP3A4 and CYP2A6 enzymes (56). NOMAC is excreted in urine and feces, likely involving Phase II metabolism. NOMAC is bound to albumin (97.5–98%) and not SHBG. The half-life of NOMAC ranges from 36.2h to 50h when administered orally alone or in combination with EE (**Table 2**).

DRSP is an active progestogen structurally nearly similar to the aldosterone antagonist spironolactone. It has a moderate affinity for the PR, a low binding affinity to the AR, with an anti-androgenic activity which is about 30% of that of CPA. Its main affinity is directed to the mineralocorticoid receptor with a strong anti-mineralocorticoid effect. It has no estrogenic or glucocorticoid activities (79). The bioavailability of DRSP is around 76% (**Table 2**). DRSP is extensively metabolized to at least 20 metabolites identified in urine and feces, including glucuronide and sulfate conjugates, and major plasma metabolites identified as an acid form of DRSP and the 4,5-dihydro-DRSP-3-sulfate (66). Some drug interaction studies involving a CYP inhibitor and inducer suggest that the metabolism of DRSP is CYP-dependent (80). DRSP is bound to albumin (95–97%) and not SHBG. The half-life of DRSP is around 30h when administered orally alone or in combination with EE (**Table 2**).

### 3.4 Drug interactions

The potential interaction of progestogens with other drugs has been the subject of numerous reports for many years. Some drug interactions involving progestogens are well documented and therapeutically relevant, but others remain unproven and/or are the subject of continuing controversy. There is strong evidence that griseofulvin (an anti-fungal drug), rifampin (an anti-tuberculosis drug), and certain anti-convulsants (phenytoin, carbamazepine, topiramate, and barbiturates) induce hepatic enzymes and reduce oral contraceptive effectiveness (81–83).

Since millions of women worldwide use hormonal contraceptives, and antibiotics are commonly used by reproductive-aged women, there has been a concern over the years regarding potential interactions between these 2 drugs, such as induction or inhibition of hepatic enzymes by either drug (82, 84). This interaction could compromise the contraceptive or antibiotic effect. However, clinical concerns regarding hormonal contraceptive-antibiotic interactions are mostly based on case reports and surveys by patients and providers, which are limited by recall bias (84). Thus, misconceptions regarding hormonal contraceptives and drug interactions are common among women, providers, and pharmacists. A majority of pharmacists recommend backup contraception for women who use antibiotics during hormonal contraception (85). It has been pointed out that such warnings may result in poor compliance with either hormonal contraceptives or antibiotics, which could increase the risk of treatment failure with either drug (82).

In a systematic review of 29 studies of drug interactions between non-rifampin antibiotics and hormonal contraceptives, it was shown that existing evidence does not support interactions between these 2 drugs (86). The study concluded that most women can expect no reduction in the effect of hormonal contraception with concurrent use of non-rifampin antibiotics.

### 3.5 Conclusions on pharmacokinetics of progestogens

Contraceptive progestins differ widely in their chemical structures. Some are structurally related to P4, others to testosterone, and one to spironolactone. These differences are reflected in their differences in metabolism, pharmacokinetics, potency and efficacy via steroid receptors, thereby confirming the absence of a class effect of the progestins.

Very few studies have been carried out on the metabolism of progestins. However, it is well known that some progestins are prodrugs and require conversion to active metabolites for biologic action. For example, several progestins are prodrugs of NET, and NGM is a complex prodrug that is converted to NGMN and LNG. Since active progestins have a Δ4,3-ketone group in ring A, they are readily converted to 3α,5α-tetrahydro metabolites, and most are then conjugated and excreted predominantly as glucuronides. A unique metabolite of NET is EE, which is formed following oral administration of NET.

More is generally known about the pharmacokinetics of progestins structurally related to testosterone than those structurally related to P4. All progestins bind weakly to albumin in blood; however, NET, LNG, ETN, and GSD also bind with high affinity to SHBG. Following their oral administration progestins such as NET, LNG, ETN, and GSD reach maximum circulating concentrations between 1 and 3 hours. The maximum concentration and area under the curve are dose dependent. Two clinically relevant pharmacokinetic parameters, bioavailability and half-life, each differ widely among the progestins. The bioavailability of NET, DSG, NOMAC, and DRSP ranges from 60-66%, whereas that of LNG, GSD, DNG, CMA, and TMG ranges from 96-100%. Similarly, the half-life of NET is very low (8 hours) in contrast to NOMAC, CPA, and CMA that have half-lives ranging from 50–80 hours.

The intracellular actions of progestins are mediated predominantly via the PR. Although progestins are designed to be potent, high-affinity PR agonists that mimic the actions of P4, a substantial number of the progestins bind to other members of the steroid receptor family, which includes the AR, MR, and GR. The progestins show considerable variation in their binding affinities via these receptors. Progestins vary widely in their reported RBAs for the AR, with some of the older generation progestins such as LNG, NET, and MPA binding with high affinity relative to testosterone, whereas TMG, DNG, and DRSP exhibit low affinities. TMG and DRSP have a relatively high affinity for the MR, whereas other progestins such as NET bind weakly to this receptor. Relatively few progestins in the significant pharmacologic range bind to the GR; a notable exceptions is GSD.

## 4 Pharmacological and clinical characteristics of progestogens for oral contraception either alone or in combination with estrogens

### 4.1 Norethisterone and norethisterone acetate

NETA is a 19-nortestosterone derivative, rapidly deacylated to NET after ingestion. Many older progestogens (NED, lynestrenol, ethynodiol diacetate and tibolone) are prodrugs of the “active” NET. This is essentially a potent progestogen with a moderate androgenic activity, like DSG and GSD, but less androgenic than LNG. All these androgenic steroids exhibit a partial agonist activity for AR-mediated transactivation (87). Moreover, as stated earlier, NET is partly converted to EE, which is negligible in contraception where NET and NETA doses are very low. COCs containing EE + a strong androgenic progestogen (e.g., EE/NETA or EE/LNG) entail a biological increase in SHBG much reduced vs estrogen combined mild or anti-androgenic progestogens (which is associated with a slight increase in free, biologically active testosterone), and changes in lipid metabolism, with slight increases in triglycerides and LDL-C, but a small decrease in HDL-C. By contrast, low doses of NET do not impair glucose metabolism (88).

Combined oral formulations currently used contain either EE 20 µg/NETA 0.5 mg (21/7 daily regimen) or an ultra low-dose of EE, 10 µg/NETA 1 mg during 24 days, followed by EE 10 µg alone for 2 days, and 2 days with inactive tablets. These formulations are effective for preventing pregnancy with safety and tolerability comparable to that reported for other low-dose COCs. With the lowest dose of EE combination (EE 10 µg/NETA 1 mg), the Pearl Index shows an acceptable contraceptive level (89). However, ultra low-dose EE/NETA displays somewhat more unscheduled bleeding/spotting per cycle, less scheduled bleeding and less tolerability than comparators such as EE 20 µg/LNG 100 µg or EE 30 µg/LNG 150 µg (90). As a POP, NET alone is used in some countries at a dose of 0.35 mg/day continuously.

In terms of thrombotic risk (venous thromboembolism - VTE and arterial thromboembolism - ATE), NETA could present a risk due to its slight conversion to EE. With use of a larger dose of NETA alone (>or= 5mg/d and > or = 90 days), the risk of VTE in a case-control study using data derived from the General Practitioner Research Database (GPRD), showed an adjusted odds ratio (OR) of 2.41 (95% CI, 1.00 to 5.78) vs non -use (91). This is a rare observation of an increased risk of VTE with high doses of NETA alone, which is not the case with the POP containing 0.35 mg of NET, as POPs do not usually increase the risk of VTE (92). However, the same POP increased the adjusted incidence rate ratio (IRR) of ischemic stroke and myocardial infarction, slightly though significantly in users, by 1.6 (95% CI, 1.2 to 2.2) and 1.5 (95% CI, 0.9 to 2.4), respectively, versus non-users (93). Moreover, low dose EE/NETA (EE ≤ 30 µg) compared to EE/LNG (EE ≤ 30 µg) in large prospective studies show a VTE risk adjusted hazard ratio (HR) of 0.73 (95% CI, 0.48 to 1.1), i.e., a risk profile roughly similar between both preparations (94).

Observation of VTE risk, which is rather similar for both EE/NETA and EE/LNG preparations, allows, rationally, to classify NETA with LNG in the so-called 2^nd^ generation of progestogens and COCs which display an important lowest risk of VTE among all combined COCs containing EE (95). However, it is noteworthy that, historically, NET was used in COCs before introduction of LNG to the market and was classified as a so-called 1^st^ generation progestogen (94).

### 4.2 Norgestrel and levonorgestrel

LNG is a potent progestogen with a stronger androgenic activity than the other androgenic progestogens used for contraception. Moreover, although having no interaction with the ER, LNG shows an anti-estrogenic action particularly at the hepatic level, decreasing the SHBG synthesis by more than 50% (i.e., ability to counteract the EE-induced SHBG production), as well as decreasing production of coagulation factors, among other anti-estrogenic actions. LNG has no glucocorticoid or anti-mineralocorticoid properties (10, 59).

The lowest gonadotropin and ovulation inhibiting dose of LNG is 60 µg/day (10). In terms of metabolism, the marked androgenic effect of LNG has a particular impact on carbohydrate and lipoprotein metabolism, showing a decrease in HDL-C and an increase in LDL-C, both changes being potentially important in terms of risk of atherosclerosis. This risk is, however, tempered by the counteracting effects of EE, in combined EE/LNG preparations. This trend is also observed, *mutatis mutandis*, in carbohydrate metabolism through increased insulin resistance, with the higher dose of LNG (250 µg/d). However, progressive reduction of both concentrations of EE and LNG, from EE 50 µg/LNG 250 µg to EE 30 µg/LNG 150 µg and then to EE 20 µg/LNG 100 µg, resulted in a decrease in the metabolic and clinical impact of these preparations (96, 97). Concerning POPs, NG 75 µg/day with continuous administration (Opill^®^), is now marketed in the USA as an OTC (over the counter, i.e., without medical prescription) with a good contraceptive effectiveness and tolerability (98). Moreover, another POP containing LNG 30 µg/d with continuous administration has been available for a long time. Both of these POPs have indeed a very low metabolic as well as vascular impact, and large studies have not demonstrated increases in VTE and ATE with these very low doses of NG and LNG (92). Moreover, LNG 1.5 mg used once within 72 hours after an unprotected intercourse, as an emergency contraception, is well tolerated and pregnancy rates are less than 3%, across different BMI and weight categories (BMI > 30 kg/m² or weight > 80 kg, respectively), with additional attention for counseling and advice for obese women (99, 100). Recently, in response to a widespread appeal among women for an “on-demand contraceptive pill”, which could be taken pericoitally (i.e., before or after sex), studies of LNG 1.5 mg showed that taken “on-demand”, these pills are efficacious, acceptable and safe (101).

Concerning combined EE 30 µg/LNG 150 µg and EE 20 µg/LNG 100 µg, which show the same ratio of EE to LNG, and are low-dose monophasic preparations with 21/7 daily regimens, review studies indicate a high contraceptive effectiveness and good cycle control, tolerability, mild or no effect on lipid and glucose metabolism, no variation in body weight/body composition among treated and untreated groups, and an incidence of adverse events comparable to those of the placebo groups. Accordingly, these combinations show an equilibrated balance between EE and LNG (102, 103).

According to numerous epidemiological studies and meta-analyses (more than 17 studies) (104), there is a consensus that use of EE ≤ 30 µg/LNG ≤ 150 µg compared with no exposure, increases the risk of VTE by about 2–3 times. It is noteworthy that EE ≤ 30 µg, combined with either LNG, NETA or NGM, i.e., 3 androgenic progestogens, show a rather similar “low” risk of VTE. Low dose EE/LNG entails the lowest risk of VTE. Consequently, the regulatory authorities of the European Medicines Agency (EMA) consider that a COC containing the lowest possible dose of EE plus a low dose of LNG should be prescribed to minimize the VTE risk (105). However, there are now studies suggesting that COCs containing natural estrogens (E2, E2V, E4) plus progestogens (NOMAC, DNG, DRSP) are safer than EE-COCs in terms of thrombotic risk, as based on results of meta-analyses (106) or through analyses of disproportionality (107).

In terms of ATE, although the absolute risks of thrombotic stroke and myocardial infarction are low in women using COCs, the relative risk for both events was increased by 1.7 (95% CI, 1.4 to 2.0) in users of EE 30-40µg/LNG vs non-users, according to a large study of Lidegaard (108), and corroborated recently (93), with ATE risks of about 2 vs non-exposure. Lidegaard hypothesized that ATE risk increased slightly with the EE content of COCs, with a relatively small impact according to progestin type.

### 4.3 Norgestimate and norelgestromin

NGM dosed at 250 µg is combined with EE 35 µg in a monophasic COC (21/7 daily regimen). Long-term trials (2 years) compared this COC to EE 30 µg combined with NG 300 µg (i.e., LNG 150 µg); they displayed the same contraceptive effectiveness, cycle control, safety, and tolerability. Moreover, in terms of lipid metabolism, NGM/EE improved levels of HDL-C and reduced LDL-C in comparison to EE/NG, while both were rather neutral in terms of carbohydrate metabolism (109). More recently, and as far as safety is concerned, NGM, despite being a less potent androgenic progestogen than LNG, was shown to robustly oppose EE regarding thrombotic risk. Indeed, meta-analyses estimated the risk of VTE with NGM/EE vs no exposure to be associated with an adjusted OR of 2.53 (95% CI, 2.15 to 3.06) vs 2.38 (95% CI, 2.18 to 2.59) for LNG/EE (110). This rather similar impact means that NGM/EE should be considered as a “so-called” 2^nd^ generation COC, as COCs containing LNG and NET are. Indeed, NGM was often classified as a 3^rd^ generation progestogen, because it was “historically” developed after LNG-COCs. However, 3^rd^ generation COCs (DSG, GSD) are characterized by a distinctly higher thrombotic risk than NGM. As far as ATE risk of oral combined EE 35 µg/NGM 250 µg is concerned, the adjusted IRR for users vs non-users is 1.9 for ischemic stroke and 2.2 for myocardial infarction, a significant-though slight-increase of specific risks. However, these are often more severe than VTE risk itself (93).

It is interesting that NGMN, being an active progestogen, is used in a contraceptive transdermal patch, at a dose of 203 µg combined with EE 34 µg of daily release. It displays a high efficacy, control of the cycle and tolerability. However, it is characterized by a definite estrogen dominance, entailing for instance a large increase in estrogen-sensitive hepatic proteins, even larger than with oral EE 35 µg/NGM 250 µg (111). This dominance may be associated with an increased VTE risk of this patch vs the oral combination EE/NGM with a OR of 1.5 (95% CI, 1.2 to 1.8) (112).

### 4.4 Desogestrel and etonogestrel

DSG was developed in order to be as potent as LNG in inhibiting gonadotropin secretion, ovarian function and endometrial development, but with a lower androgenic potency in order to limit the androgenic impact on lipids. DSG 150 µg is combined with EE, either 30 or 20 µg in a monophasic 21/7 daily regimen, while another biphasic, estrogen dominant combination shows a first 7-day phase with higher EE concentration (40 µg) combined with lower DSG (only 25 µg) and a second 15-day phase with EE 30 µg/DSG 150 µg (22/6 daily regimen). These combined preparations are effective oral contraceptives with high tolerability and general safety (113). Indeed, the DSG metabolic impact shows increased HDL-C and Apo-A, and somewhat decreased LDL-C; this is a significantly improved pattern when compared to low-dose preparations, either monophasic EE 30 or 20 µg/LNG 150 or 100 µg, or triphasic OCs with a EE to LNG ratio slightly more estrogenic (114). Moreover, studies of glucose metabolism showed minimal changes in plasma glucose, insulin and C-peptide with use of these low-dose EE/DSG COCs (115, 116).

In the comparison between EE/DSG vs EE/LNG, and despite diverse studies showing some mild changes in individual coagulation factors indicating some increases in procoagulant and decreases in anti-coagulant factors with both types of COCs, it was first considered that the risk of VTE was similar for both preparations. In contradistinction, early analysis by Kemmeren et al. showed that both DSG and GSD preparations (so called 3^rd^ generation COCs) presented an adjusted OR vs 2^nd^ generation LNG-COCs of 1.7 (95% CI, 1.4 to 2.0), which was significantly increased, in disfavor of DSG and GSD products (117). Thereafter, numerous meta-analyses confirmed this observation (104, 118). It is still debatable whether this higher risk of VTE for 3^rd^ generation OCs is attributable to lower androgenic potency than for 2^nd^ generation OCs to oppose the stimulatory effect of EE on coagulation (119). However, in terms of hemostasis, the global test of activated protein C resistance shows that DSG products are more procoagulant than the LNG ones (120). DSG alone, i.e., deprived of any added estrogen, used at 75 µg per tablet continuously, is largely used as a contraceptive with no increased VTE risk (92). Moreover, ENG, the active form of DSG, is largely used in a flexible vaginal ring releasing a daily dose of 15 µg EE and 120 µg of ENG and also used as a subdermal implant (68 mg per implant, with replacement every 3 years), and is considered as an effective long-acting reversible contraceptive (LARC) (121).

In terms of ATE, oral combined EE/DSG OC use vs non-use, increase slightly, though significantly, ischemic stroke and myocardial infarction risks from 1.3 to 2.5 according to studies, essentially in proportion to the estrogen dose (93, 108, 122). A very slight increase in both risks was observed by Yonis in users of oral DSG alone, which could be a bias in the selection of patients at higher risk receiving POP contraception (93). It is noteworthy that pre-existing risk factors (age, hypertension, CVD…) also facilitate occurrence of ATE in OC users (123).

### 4.5 Gestodene

GSD has a strong PR affinity and efficacy, leading to potent ovulation inhibition (lower inhibitory dose is 40 µg, while it is 60 µg for LNG, and 1 mg for CPA), and to a more potent inhibition of endometrial proliferation than with LNG (10, 124). Accordingly, its combination with EE 30 or 20 µg is the lowest dose of any progestogen in a COC, with 75 µg. Although GSD has a high affinity for the AR, its androgenicity is moderate, potentially due to GSD inhibition of hepatic 5alpha-reductase, and the GSD ratio of progestogenic to androgenic effects is higher than the LNG corresponding ratio (125). Moreover, in COCs containing the same dose of EE, GSD does not decrease SHBG as much as LNG, which is a more androgenic progestogen (126). GSD does not bind to ER and, by contrast, has a relatively high affinity for the mineralocorticoid receptor, but a very weak anti-mineralocorticoid effect (76).

GSD-containing COCs are EE 30 µg/GSD 75 µg, EE 20 µg/GSD 75 µg, both monophasic (21/7 daily regimen), EE 15 µg/GSD 60 µg, monophasic (24/4 daily regimen), and a triphasic (21/7) 30/50-40/70-30/100 µg. They all show a high contraceptive effectiveness and cycle control (more unscheduled bleeding/spotting with the lowest 15/60 combination), as well as a good tolerability (77). GSD-containing COCs show a low impact on lipid metabolism with small increases in HDL-C and decreases in LDL-C but an increase in triglycerides, due to a slight estrogen dominance of these preparations. Nevertheless, all these changes remain within the normal range (127). COCs containing GSD revealed only minimal effects on insulin, C-peptide and plasma glucose, but a study of 13 cycles of use of EE/GSD showed a trend to increase fasting glucose by 10-12% vs baseline, although these values also remained within the clinical normal range (128).

Hemostasis has been studied during use of EE/GSD combinations (76). Increases in procoagulant and decreases in anti-coagulant individual factors, and studies of markers of coagulation and fibrinolytic activities together showed a trend in excess risk of coagulation. More specifically, global tests like endogenous thrombin potential (ETP) – based activated protein C resistance show substantial increases in coagulation risk for GSD and DSG, both members of the so-called 3^rd^ generation of EE-COCs (18). Epidemiological data are congruent with global hemostasis tests: EE/GSD use increases the risk of VTE vs non-use and vs EE/LNG used as a classical comparator. The adjusted OR of EE/GSD vs non-exposure is 3.64 (95% CI, 3.00 to 4.43), and vs EE/LNG, the relative risk is 1.52 (95% CI, 1.24 to 1.87) (110). These increases in risk of VTE are in the same range for EE/GSD and for EE/DSG, indicating that this risk is significantly more elevated (by 1.5–2 times) for the so-called 3^rd^ generation than for the 2^nd^ generation COCs. In addition to these congruent results, a disproportionality analysis of VTE events associated with various COCs formulations also shows that the reporting ratio for EE/LNG indicates a lower risk (0.28) than for EE/GSD (0.48) or for EE/DSG (0.52) (107).

Finally, it should be emphasized that these studies have not investigated separately the VTE risk of GSD combinations with EE 15, 20 or 30 µg for a potential difference in risk, particularly with the lowest EE dose (15 µg) in combination with GSD. By contrast, ATE risk, in users vs non-users of oral COCs containing GSD 75 µg with either EE 30 µg or 20 µg was significantly increased in the same range for both ischemic stroke and myocardial infarction, with an adjusted IRR between 1.2 to 2.0 with the EE 30 µg OC, and about 10% less with the EE 20 µg OC (93, 108).

### 4.6 Dienogest

As the 17-alpha ethinyl group protects nortestosterone progestogens from CYP enzyme-dependent degradation, DNG (without the ethinyl group) has to be administered in doses of one or more milligrams to be effective. At these dosages, DNG displays a strong progestogenic activity on the endometrium with a transformation dose of 6.3 mg per cycle, similar to that of LNG. The presence of the double bond in the steroidal B-ring may partly increase the PR affinity of DNG (129). Although not powerfully anti-gonadotropic, DNG is anti-ovulatory with a threshold dose of 1 mg, in the same order as CMA and CPA (48). DNG is the only non-androgenic 19-nortestosterone derivative – and, by contrast, an anti-androgenic compound whose effects appear AR-mediated (1). Its anti-androgenic activity is about 40% of that of CPA. Accordingly, when combined with EE, its action increases HDL-C, triglycerides and SHBG concentrations (87) and, altogether, it does not antagonize the estrogen induced alterations of certain hepatic proteins, like coagulation factors (10). DNG has no estrogenic, glucocorticoid or mineralocorticoid activity by itself.

There are numerous clinical uses of DNG, particularly because of its potent endometrial anti-proliferative action, its absence of androgenic effect, and its rather neutral metabolic impact including a limited hepatic effect. Therefore, DNG is used alone (2 mg daily) in endometriosis (130). It is noteworthy that already in 1995 (48), it was envisaged to use DNG for contraception, in combination with E2, based on its potent endometrial action, at a time when other combinations of E2 plus different progestogens had failed dramatically, because of insufficient cycle control and hemorrhage (131).

DNG was originally combined with EE (EE 30 µg/DNG 2 mg, monophasic 21/7 daily regimen), marketed since 1995, and later, in 2009, combined with E2V in a four-phasic “dynamic dosing” preparation with the following regimen: 2 days 3 mg E2V/5 days 2 mg E2V + 2 mg DNG/17 days 2mg E2V + 3 mg DNG/2 days 1 mg E2V and 2 days of placebo (26/2 daily regimen), i.e., a regimen with a high concentration of estrogen at the beginning of the cycle with a stepdown thereafter, while DNG showed a long, increasing, progestogen step-up. Their combination entailed an early proliferation of the endometrium, with a subsequent strong anti-proliferative action. Both COCs were similarly effective in contraception, and treatment with E2V/DNG when compared to EE/DNG resulted in milder pituitary downregulation and reduced hepatic SHBG synthesis (132). Control of the cycle, probably due to the four-phasic disposition, was almost similarly effective with both COCs, with a low level of unscheduled bleeding, and a lower volume of bleeding/spotting with E2V/DNG, which became an indication for treating heavy menstrual bleeding (HMB) (133). However, incidence of scheduled (withdrawal) bleeding with this latter COC occurred in ~75% of cycles, i.e., 15 to 20% less than with EE/DNG use (134).

In terms of metabolic impact on carbohydrate and lipid profile, both COCs were essentially neutral, with EE/DNG showing a slightly more estrogenic action than E2V/DNG with no clinically meaningful differences. However, when compared to EE/LNG, both DNG OCs increased HDL-C and decreased LDL-C more effectively, showing a classical difference between COCs containing an anti-androgenic progestogen (DNG) or an androgenic progestogen (LNG) (135, 136). Hormonal studies of both DNG-COCs showed a classical pituitary-ovarian fully reversible inhibition of gonadotropins, estrogens, anti-Müllerian hormone (AMH), total and free testosterone, which were more decreased, while SHBG was more increased by EE/DNG, with a pattern more favorable for treating polycystic ovary syndrome (PCOS) than E2V/DNG (132).

Regarding effects on the serum proteome, mainly originating from hepatic protein synthesis (but also from metabolism, inflammatory processes, etc.), comparison of EE/DNG to E2V/DNG showed impressive differences. After 9 weeks of use of these COCs, the number of proteins/protein families detected and quantified were 121 for EE/DNG, 5 for E2V/DNG and no change for DNG alone: The complement system, metabolism, and coagulation were the most affected functions under EE compared to E2V (137).

As far as coagulation is concerned with EE/DNG use, it was progressively apparent through large epidemiological Post Authorization Safety Studies (PASS), and other studies, that, as classified by the EMA, the estimated risk of VTE events associated with COCs was 5–7 events/10,000 women per year (vs 2/10, 000 WY for non-use) for COCs containing EE and LNG, or NGM (2^nd^ generation COCs), or NETA (1^st^ generation COCs). COCs containing EE + DSG or GSD were associated with a risk of 9 -12/10,000 WY (3^rd^ generation OCs) and the same risk was also delineated for EE +DRSP (and also for CMA and CPA (cited with DRSP as so-called 4^th^ generation OCs) (105). As an update to the EMA list (138), risk of VTE with COCs containing EE/DNG was estimated to be 8-11/10,000 WY. This would allow classification of EE/DNG as comparable for thrombotic events to the 3^rd^ - 4^th^ so-called generation of OCs. Accordingly, the VTE risk of EE/DNG shows a two-fold increase when compared with EE/LNG, with an adjusted OR of 2.26 (95% CI, 1.80 to 2.84) (118).

It was recently suggested that a prolonged-release (PR) formulation of EE 20 µg – DNG 2 mg preparation in a 24 + 4 daily regimen would offer an improved bleeding profile and a minimal impact on coagulation (139, 140). However, critical analysis of this data highlights discrepancies between published and regulatory data on the bleeding profile, rendering the potential improvement unclear. Moreover, the clotting time (aPTT) based-APCR is inappropriate for detection of contraception-induced hypercoagulability. Another recognized method (thrombin generation assay; ETP-based nAPCsr) should be essential to perform before asserting a neutral or favorable safety profile to the new PR-EE/DNG preparation. Finally, the high incidence of VTE events during clinical trials of this PR-preparation yields a residual incidence rate of 34.1 (95% CI, 13.4 to 71.7) per 10,000 WY, contradicting any improvement of the coagulation profile (141).

The outcome in terms of risk of VTE is quite different for E2V-DNG: a pooled data analysis of two large, prospective, observational cohorts compared the risk of VTE in over 11,000 E2V/DNG users vs over 18,000 EE/LNG users. A significantly decreased risk for E2V/DNG vs EE-LNG was observed (propensity score-stratified), with an adjusted hazard ratio (HR) of 0.46 (95% CI, 0.22 to 0.98) (142). Moreover, in a systematic review and meta-analysis of COCs containing either natural estrogens (E2, E2V) or synthetic EE, and conducted in 5 relevant studies involving over 560,000 women, a significant 33% reduction in VTE risk was demonstrated among users of natural estrogen-based COCs (E2V/DNG and E2/NOMAC) compared to EE-based COCs (OR = 0.67 (95% CI, 0.51 to 0.87). When considering studies allowing adjustment for confounding factors (e.g. age, smoking status, family history of VTE…), an even 49% lower risk of VTE was reported for E2-based COCs vs the gold standard, i.e. EE/LNG. Both E2-containing OCs showed a rather similarly decreased OR vs EE-based COCs (106).

In conclusion, DNG conveys potent progestogenic and moderate anti-androgenic effects. Its combination with EE results in a conventional COC resembling 3^rd^-4^th^ generation OCs, with excellent contraceptive effectiveness and tolerance, but with a persisting VTE risk higher than the one of EE/LNG. With similar effectiveness and tolerability, E2V/DNG displays a much lower risk of VTE than the purported “gold standard” EE/LNG.

### 4.7 Chlormadinone acetate

CMA alone, used in large doses of 10 mg/day for 20 days per month and for a mean of 33 months, showed a full contraceptive effect with inhibition of the hypothalamic-pituitary-ovarian axis (143). When combined with EE, CMA anti-gonadotropic action is readily obtained with 1.5–2 mg daily to ensure inhibition of ovulation (144). CMA induces the transformation of the estrogen-primed endometrium, and even when combined with a high dose of EE, such as 50 µg, CMA 2 mg was not associated with endometrial hyperplasia (145). Among specific properties of CMA, its anti-androgenic activity, corresponding to 20% of that of CPA, was evident (10). Numerous studies, whether non-comparative, or with comparison to EE/LNG, or compared to placebo, showed a non-inferiority vs comparator, of the CMA-containing preparations (EE 30 or 20 µg/CMA 2 mg) for the treatment of acne, seborrhea, alopecia and mild hirsutism, with a decrease, for example, of about 50% or more in facial papules and pustules (50).

In terms of clinical effects, CMA was mostly used in Germany and Switzerland since the end of the 1990s for contraception and skin androgenic symptoms under the formulation of EE 30 µg/CMA 2 mg, monophasic, 21/7 daily regimen, and later EE 20 µg/CMA 2 mg, monophasic, 24/4 daily regimen, but also as a progestogen alone, whether as a hormonal contraception at 10 mg/day in the case of estrogen contraindication, or as a treatment of uterine benign pathology (myomas, endometriosis, organic uterine bleeding, etc.). As a COC, EE/CMA shows good cycle control, high contraceptive effectiveness, efficient acne resolution, weight and blood pressure unchanged, and good tolerability (144). The impact on metabolism of these COCs, whether containing EE 30 or 20 µg, was limited and showed a slight though definite estrogen action probably facilitated by the non-androgenic nature of CMA. Indeed, study of lipid metabolism showed an increase in triglycerides, HDL-C, VLDL-C, apolipoproteins AI and AII, and a decrease in LDL-C, most of the time significant though essentially keeping ascribed to the normal range, in agreement with the slight estrogenic effect of EE/CMA. Carbohydrate metabolism was also kept within normal limits as far as plasma glucose, insulin and C-peptide were concerned, and there was no trend to impaired glucose tolerance with these COCs (146, 147).

The hemostatic study showed the same impact for both EE/CMA preparations and for the comparator EE 20 µg/DSG 150 µg, with increased pro-coagulation factors, decrease in main natural anti-coagulants and increase in fibrinolysis parameters, indicating a potential trend to increased thrombotic risk. More interesting, Winkler et al. also compared the EE/CMA COCs to EE 30 µg/LNG 150 µg and found for the latter COC less increase in procoagulant and less decrease in anti-coagulation than with EE/CMA. Moreover, they used the global ETP based-APC resistance (APCr) test, and their results showed that EE 30/LNG150 displayed a significantly lower APCr value, indicating a lower risk of thrombosis than EE/CMA and EE/DSG (146, 147). Analysis in 2014 by Ziller et al. of a German gynecological database, computerized 68,168 contraceptive users (148). The adjusted risk of VTE in EE/CMA users vs users of LNG-COCs showed an OR of 2.54 (95% CI, 0.72 to 9.04). This study suffered from numerous limitations, including a low number of EE/CMA users. More recently, the nested case-control analysis using German Claims data for risk of VTE (118), clearly showed that the thrombotic risk was increased two-fold for COCs containing EE and CMA, when compared to EE/LNG users, with an adjusted OR of 2.06 (95% CI, 1.58 to 2.68). Once again, it was interesting to note, in the same study, that analyses of COCs containing EE confirmed that EE/LNG was associated with the lowest risk of VTE. Indeed, EE/CMA, with a risk two times higher than EE/LNG COCs could obviously be ranked in the same category of VTE risk as DNG-, CPA-, DSG-, GSD- and DRSP-containing EE-COCs (118).

Finally, CMA is also used alone, either when EE is contraindicated for contraception, or to treat diverse gynecological, mainly uterine, pathologies. However, in these cases, CMA is often used at 10 mg daily, a dose well tolerated (143), but usually for long periods of time (up to several years) and with no or very limited cardiovascular risk, an advantage of POPs (92). However, long-term and high dosages of CMA, CPA, and NOMAC, all three anti-androgenic progestogens, are associated with a possibility of developing meningiomas (149), which is not the case for contraception, as COCs only contain smaller dosages of these progestogens.

### 4.8 Cyproterone acetate

The CPA effective oral dose for inhibition of ovulation and for full transformation of the endometrium is 1 mg/day (59). Clinical indications of CPA high-dose, alone or combined with EE are essentially based on its anti-androgenic and progestogenic properties. CPA-alone is used at a high dose (25–100 mg/day) in men for hypersexuality, sexual deviations, and as advanced deprivation therapy (ADT) for prostate cancer (an IM form is available). In women, CPA-alone is also used in severe problems of non-tumoral hyperandrogenism, such as severe hirsutism, complicated acne, and alopecia. CPA 25 mg/day or 50 mg/day induces amenorrhea because of hypothalamic-pituitary profound inhibition, fat tissue accumulation, prolonged release and a long half-life. In order to restore a withdrawal bleeding, high dose CPA is used with EE, e.g., 35 or 50 µg in a reverse sequential regimen (“Hammerstein regimen” (150),, with CPA (≥ 25 mg/day) from day 5 to 14 of the cycle and EE day 5 to day 24 or better for safety, replacing EE by oral E2 or transdermal E2 (151, 152). Contraceptive effectiveness and improved general tolerance (including hepatic tolerance) to these high doses are obtained although fatigue and loss of libido are frequent. However, duration of treatment should be limited to some months (except for prostate carcinoma), because of the association of high doses of CPA with meningiomas (see later), and of thrombotic risk due to its combination with EE. Moreover, if high dose CPA is not taken scrupulously regularly and a pregnancy occurs, feminization of a male fetus could occur.

CPA 2 mg combined with EE 35 µg, monophasic 21/7 (daily regimen Diane 35^®^), is marketed for treatment of signs of androgenization, acne and seborrhea in women, particularly if they already used another effective contraception, to which CPA or EE/CPA is adding a notable anti-androgenic effect. However, full studies of EE/CPA contraceptive effects *sensu stricto* have not been made by the marketing authorization holder (MAH), although extensive reviews and long use (> 50 years) of EE 50 µg and later EE 35 µg/CPA 2 mg have shown contraceptive effectiveness, good cycle control and tolerability (153).

CPA effects on metabolism are largely neutral in relation to its pregnane structure (and in opposition to androgenic progestogens). When CPA is used in low-dose EE/CPA combination, it displays an estrogenic pre-eminence which translates in lipid metabolism by increases in HDL-C, triglycerides, and decreases in LDL-C, while carbohydrate metabolism is only slightly affected by some increase in insulin resistance in users of high-dose CPA (25–100 mg/day). The impact of low-dose EE/CPA is neutral in studies using glucose oral challenges (OGTTs) (154, 155). In a study using the euglycemic hyperinsulinemic glucose clamp technique, our group showed an unaltered peripheral (presumably muscular) insulin sensitivity and a slight increase in insulin (presumably hepatic) clearance at one-year use of EE 35 µg/CPA 2 mg, underlining the metabolic moderate effect of this COC on the liver (156).

Numerous trials were devoted to the study of the anti-androgenic effects of the low-dose EE/CPA combination (157). A Cochrane review of COCs and treatment of acne reported that low-dose EE/CPA improved acne better than COCs containing EE/DSG or LNG, but OCs containing CMA were equally effective vs EE/CPA (158). Concerning hirsutism, another Cochrane collaboration study concluded that EE/CPA resulted in subjective improvement of hirsutism compared to placebo, but a better outcome with CPA vs other medical therapies (e.g., ketoconazole, spironolactone, etc.) was not demonstrated (159).

EE/CPA has been indicated for treatment of acne and/or hirsutism, which are symptoms in PCOS: PCOS is characterized by hyperandrogenism, polycystic ovaries identified by ultrasound, and oligomenorrhea (160). Importantly, PCOS is frequently associated with cardiovascular disease (CVD) events, and this association is mediated by obesity and metabolic syndrome conditions, already in women aged ≤ 40 years (161). First line treatment by COCs, particularly of the so-called 4^th^ generation, EE combined with CMA, DRSP or CPA, is selected because these OCs are metabolically neutral and regulate the cycle, but also largely inhibit the increased androgens (mainly free and total testosterone, DHT, and androstenedione) (5, 162, 163). However, Glintborg et al. showed that in PCOS, thrombin generation, which is an important risk marker of CVD and reflects the effects of multiple markers of coagulation, is significantly increased (P< 0.01) vs controls, and is stimulated by EE (164). This important observation is instrumental for choosing COCs which do not increase endogenous thrombin potential-based activated protein C resistance and associated thrombotic risk. Accordingly, COCs not containing EE, but rather less potent estrogens, E2, E2V or E4 such as E2/NOMAC, E2V/DNG and E4/DRSP have been interestingly shown to entail lower levels of thrombin generation and ETP-based APCr (165). Moreover, these natural estrogen-containing COCs also inhibit the gonadotropin-ovarian axis, contain an anti-androgenic progestogen, effectively control the cycle, block the androgens, and are essentially metabolically neutral. Obviously, for all these reasons, the natural estrogen-containing COCs should be candidates for the endocrine management of PCOS (106, 162, 166, 167).

After many discordant results of studies concerning the risk of EE/CPA, meta-analyses are now consensual and show that this risk is in the range of the other COCs containing either mild androgenic progestogens (DSG, GSD) or antiandrogenic progestogens (DNG, CMA, CPA, NOMAC, DRSP). Indeed, low-dose EE/CPA users show an adjusted OR of 4.27 vs non-treated women and of 1.80 (95% CI, 1.49 to 2.18) vs users of EE/LNG (110). This means that EE/CPA increases by 14 extra cases the number of VTE per year/10,000 treated women – a range also found for DSG, GSD, DNG, CMA, NOMAC and DRSP contained in EE-COCs. These results close a discussion initiated in 2013 by the French Medicine Agency (ANSM), requesting to suspend marketing authorization for Diane 35^®^ for excess thrombotic events and extensive off-label use of this “COC” as a contraceptive only. A Europe-wide review was initiated by EMA and concluded that the benefits of Diane 35^®^ outweigh the risks [among which the risk of VTE incurred during pregnancy and post-partum] (168). However, the EMA’s Pharmacovigilance Risk Assessment Committee (PRAC) recommended that “Diane-35 ^®^” is indicated for the treatment of moderate to severe acne related to androgen-sensitivity (with or without seborrhea) and/or hirsutism in women of reproductive age”, and it recommended to increase awareness about its risk of VTE and strengthened the warning and precautions regarding the risk (105).

The use of three specific progestogens (2 pregnane-acetate CPA and CMA, and one norpregnane-acetate, NOMAC) have been - until now - associated with an increased risk of developing meningiomas. It occurs essentially when these progestogens are used for a long-term and at high dose (169). This is particularly the case for CPA used as well for men and for women for different indications bound to hyperandrogenism, sexual disorders and prostate cancer, as discussed earlier. In women, specific indications are concerned not only with severe hyperandrogenism, but also endometriosis, fibromyomas and other disorders with long-term doses of 10 to 100 mg/daily. By contrast, for contraception and skin disorders, use of the low-dose monophasic EE 35mcg/CPA 2 mg is not reported, even when taken for a very long time, to be associated with development of meningiomas (169). Use of high dose CPA (i.e., higher than a total of 3gr of CPA taken during the first 6 months of treatment) increases the risk of incidence of meningiomas to 23.8/100,000 person-years, compared to a general population incidence of 8 cases per 100,000 person-years (170). EMA contraindicates use of CPA (and also CMA and NOMAC) in women already bearing a meningioma and advises to avoid long-term high doses of these progestins without a close surveillance (149).

### 4.9 Nomegestrol acetate

NOMAC is a potent progestin without concomitant estrogenic or androgenic activity. It strongly inhibits gonadotropin secretion and ovarian function, induces ovulation inhibition with a daily dose of 1.25 mg, and entails endometrial secretory transformation. NOMAC is also an anti-androgen with a potency of about 20-30% of CPA anti-androgenicity, in between CMA (20%) and DRSP (30%) (15). It has no glucocorticoid or anti-mineralocorticoid effects.

Clinical oral administration of NOMAC alone is frequently used (5 mg up to 10 mg daily, either continuously or sequentially) for cycle disorders (e.g., benign unscheduled bleeding, dysmenorrhea, etc.) or fibromyomas and endometriosis (56). Continuous administration of NOMAC with doses as low as 2.5 mg are effectively contraceptive. NOMAC alone is not considered to increase thrombotic risk (92, 171). However, definitive demonstration has not been reached, as a suspicion was raised that norpregnanes, including NOMAC, could interfere with APCr (172). NOMAC alone (5 mg/day) did not affect levels of total cholesterol, HDL-C and LDL-C and even transiently decreased triglycerides (171), and did not induce changes in plasma glucose, insulin and HbA1C (173).

A hormonal combination of NOMAC 2.5 mg with E2 1.5 mg, monophasic (24/4 daily regimen) was introduced in the EU in 2011, to potentially minimize the cardiovascular risk observed with COCs containing EE (174). Studies of E2 1.5 mg/NOMAC 2.5mg showed that control of the cycle was quite acceptable with this monophasic COC displaying a withdrawal bleeding of 2–3 days, lighter than with the comparator EE 30 µg/DRSP 3 mg (21/7) associated with menses of 3–4 days duration. However, scheduled withdrawal bleeding with E2/NOMAC was progressively less frequent with amenorrhea ranging from 22% (cycle 4) to 31% (cycle 12), whereas it was 3 to 6% with EE/DRSP. Unscheduled bleeding/spotting was similar for both OCs, decreasing from 20 to 11% as a function of time (175, 176). Contraceptive effectiveness of E2/NOMAC showed a Pearl Index of 0.31 (95% CI, 0.08 to 0.79) for women 18–50 years of age (176). This was confirmed later in a large PASS study (177): E2/NOMAC demonstrated a superior contraceptive effectiveness compared to LNG-COCs, likely due to the comparatively short hormone-free interval (24/4 for the former vs 21/7 for the latter), possibly reinforced by the longer half-life of NOMAC.

In terms of metabolic effects, E2/NOMAC does not clinically significantly alter lipid and glucose metabolism, whereas EE/LNG used as a comparator can alter cholesterol levels and insulin sensitivity (178, 179). Tolerability is excellent for E2/NOMAC with almost no change of weight during use, and no change in blood pressure. Continuation rates and satisfaction are also high (180, 181). In terms of control of the cycle, the absence of withdrawal bleeding in more than 20% of women and cycles, which was verified, was perhaps felt by the users either as a relief or as somewhat anxiogenic, although contraceptive effectiveness was high.

Two extensive studies of hemostasis were conducted in 2011, one during 3 cycles of E2/NOMAC vs EE 20 µg/LNG 100 µg (179) and the other during 6 cycles of E2/NOMAC vs EE 30 µg/LNG 150 µg (178). In both studies, the impact of E2/NOMAC was very limited on individual parameters of coagulation, anti-coagulation and fibrinolysis, whereas for both EE/LNG preparations, these hemostasis factors were significantly altered. However, the important global assay of endogenous thrombin potential based-activated protein C resistance (ETP based-APCr) conducted with the authors own methods of assessment in both studies, showed a significant lower resistance to APC than with EE/LNG, which potentially indicates also a lower risk of VTE for E2/NOMAC in spite of EE/LNG being considered as associated with the lowest risk of thrombotic events (95, 182). A comparative PASS study of E2/NOMAC vs EE/LNG (PRO-E2 study) was conducted on a large scale to investigate whether E2/NOMAC was at least non inferior to EE/LNG in terms of thrombotic risk. This study was conducted in 49,598 E2/NOMAC users and 51,900 COC-LNG users followed up for two years. The adjusted HR of the former vs the latter was 0.59 (95% CI, 0.25 to 1.35) for the primary outcome related to DVT of the lower extremities and PE. Overall, the analyses conducted in this study showed that the VTE risk - and also the ATE risk - was not higher with E2/NOMAC than with LNG-COC use (183). Moreover, a recent meta-analysis conducted according to the PRISMA guidelines compared the VTE risk of COCs containing natural estrogens E2V/DNG and E2/NOMAC vs EE-containing COCs. The OR of the natural estrogen-COCs vs synthetic EE-COCs was 0.67 (95% CI, 0.51 to 0.87), i.e., a 33% significant reduction in VTE risk (and a 49% adjusted reduced VTE risk), in favor of natural estrogen-containing OCs (106). Additionally, Didembourg et al. conducted a disproportionality reporting rate analysis of VTE events associated with various COCs by extracting individual case reports from the Eudravigilance database (107). They particularly compared reporting rates of E2V/DNG, E2/NOMAC and E4/DRSP vs EE-containing COCs, including EE/LNG. Specifically, E4/DRSP showed the lowest reporting rate of 0.07 (similar to POPs, including DRSP-alone). This rate was 0.13 for both E2V/DNG and E2/NOMAC, whereas all EE-COCs had a significantly higher disproportionality reporting rate. Comparison of natural estrogen-COCs vs EE/LNG was in favor of all three natural estrogen-based COCs (P value of the comparison, <0.001).

Finally, as it has been established earlier that a reduced sensitivity to APC is an independent risk factor of VTE (182), the group of Douxfils and Morimont used a standardized and validated ETP based-nAPCsr method (184) for assessing APCr of users of COCs containing natural estrogens or EE. They constructed by interpolation an exploratory model using nAPCsr values on an X axis vs VTE risk of COCs established by de Bastos (95) on a Y axis; a strong correlation between these variables was obtained: a low level of nAPCsr for E2/NOMAC (2.57) corresponded to a low level of VTE risk, while by comparison, EE 30 µg/LNG with a nAPCsr of 3.51 corresponded to an intermediate VTE risk, and EE 30 µg/DSG with a nAPCsr of 5.41 corresponded to a distinctly higher risk of VTE. This exploratory model showed that nAPCsr values may reflect the prothrombotic profile of COCs and that a natural estrogen-containing COC (E2/NOMAC) is associated with a lower thrombotic risk than EE-COCs, including a lower risk than EE-LNG (whether containing 20 or 30 µg of EE) (185).

NOMAC is considered to increase the risk of meningiomas as a function of length of time of use and cumulative doses (169). When NOMAC is used at low dose (as in E2/NOMAC), the incidence of meningiomas is near to no use, at 7.0 per 100,000 person-years. High cumulative doses of NOMAC (>6 gr) have been described with an incidence of meningiomas in users of 91.5 per 100,000 person-years, a 13-fold increase compared to non-users in the study of Nguyen et al. (186). If high doses (5–10 mg/day) are chronically administered, occurrence of headache or other neurological symptoms should prompt a clinical investigation.

### 4.10 Drospirenone

Whereas estrogens (e.g., EE, E2) activate the renin-angiotensin-aldosterone system (RAAS) leading to sodium and water retention through the effect of aldosterone at the renal tubule, this effect is counteracted by the potent anti-mineralocorticoid action of DRSP (already at low dosages of 1–3 mg/day) leading to sodium excretion and potassium sparing (without hyperkalemia, or very rarely [~0.2% of users]) (187). In compensation for this rise in sodium excretion, plasma renin activity is increased, including a 65% increase in aldosterone levels (188).

DRSP has a moderate binding affinity to the PR. However, this intermediate activity of DRSP can inhibit gonadotropin secretion and ovulation with 2 mg/day. Its transformation dose of the endometrium is 50 mg/per cycle (59); Its binding affinity to the AR allows DRSP to develop an anti-androgenic activity of about 30% of that of CPA, and, in so doing, DRSP has become the most used anti-androgen in the United States for many years. It has no estrogenic nor appreciable glucocorticoid effect (60).

In terms of PK (see details in PK Part), the bioavailability of DRSP is 63%, with a long elimination half-life of 36.2 to 50 hr, with 95-98% bound non-specifically to albumin and no binding to SHBG or CBG. According to these characteristics, DRSP tends to accumulate in blood during multiple dosing, which could be useful in case of missed pills for prolonging contraceptive effects (189).

DRSP, as an anti-androgenic, anti-mineralocorticoid progestogen, has been classified as a “so-called” 4^th^ Generation representative, and largely clinically used either as DRSP alone, 4 mg, in a 24/4 day regimen (as a POP), or as a COC combining EE 30 µg/DRSP 3mg monophasic in a 21/7 regimen, or a COC composed of EE 20 µg/DRSP 3 mg in a 21/7 regimen, and also in a 24/4 days regimen. Moreover, recently, a COC containing E4–15 mg as a monohydrate/DRSP 3mg monophasic, 24/4 days regimen, was introduced to the market in 2021.

DRSP alone, non-micronized, is dosed at 4 mg, in a 24/4-day regimen, and not continuously administered as other POPs, to, hopefully, reduce unscheduled bleeding and improve control of the cycle (190). It effectively inhibits gonadotropins and ovarian activity (191), and accordingly its contraceptive effectiveness (Pearl Index 0.73 in European studies and 4.0 in US studies) is in the range of combined formulations. No VTE was detected in 2500 women followed during 25,000 evaluable cycles, a usual observation with POPs (92, 192). There was no difference in contraceptive efficacy between obese and non-obese users, and the same high level of safety and tolerability was also observed (193). Moreover, when comparing DSG used continuously to DRSP, both POPs inhibited ovulation effectively, despite the 4-day hormone-free interval for DRSP-alone; anovulation was still maintained with DRSP-alone, even when multiple intentional 24h delays in tablet intake were performed (191). An explanation of this effectiveness may be found in the long half-life of DRSP (36 to 50 hours). However, DRSP-alone will be metabolized more than when associated with EE, because EE effectively inhibits CYP metabolic enzymes (194).

As control of the cycle is a key feature of COCs, analysis of bleeding patterns in users of DRSP alone showed that scheduled bleeding (as DRSP has a 24/4 cyclic dosing) decreased from 81.2% at baseline to 26.4% in cycle 12, and unscheduled bleeding from 54.4% to 41.6% in cycle 12 (134). When compared to the DSG containing POP, less bleeding and discontinuation rates were observed in DRSP-only users (195).

The metabolic impact of DRSP alone compared with the DSG POP over 9 months of use entailed only small changes in all lipid fractions. The DRSP-only effect on lipid parameters could be considered as neutral and the same also applied to the study of glucose metabolism. Hemostatic analyses of some major parameters of coagulation essentially showed a median reduction in D-dimers levels by 17.8%, which did not suggest a pathological trend. Indeed, there was no VTE observed with use of DRSP alone in all performed trials (196, 197). As being a progestogen-only contraceptive, DRSP alone was not submitted to an Agency request for a PASS study, because oral progestogens alone are not reported to increase the risk of VTE (92). It is, however, interesting to know that in a recent large disproportionality reporting rate analysis of the Eudravigilance database bearing on most COCs, DRSP alone was also analyzed and presented the lowest score of VTE risk, altogether with E4/DRSP, whereas EE/DRSP obtained a very high score of VTE risk (107).

As DRSP through its anti-mineralocorticoid action is reducing RAAS, including aldosterone effects, it is of interest that although DRSP alone did not interfere with blood pressure in normotensive users, it was able to slightly reduce DBP (diastolic blood pressure) and SBP (systolic blood pressure) in women with high normal BP (i.e., DBP between 85 to 90 mm Hg and SBP 130 to 140 mm Hg) (192, 198).

The three combinations of DRSP + EE comprise EE 30 µg/DRSP 3 mg - 21/7 days regimen: EE 20 µg/DRSP 3mg - 21/7 days regimen, and EE 20 µg/DRSP 3mg - 24/4 days regimen. These 3 monophasic COCs were marketed in the beginning of 2000s. PK characteristics of these 3 formulations are near to that of DRSP alone. However, as EE is an inhibitor of CYP 450 metabolizing enzymes, this results in higher plasma concentrations of DRSP with EE/DRSP formulations than with DRSP alone (194). Contraceptive effectiveness is high with a Pearl index between 0.2 and 0.5 according to different studies and, in a large cohort study (189), the rate of contraceptive failure was 2.1% with EE/DRSP 24/4, slightly better than the rate of the two other EE/DRSP formulations and also of other COCs. This is probably due to the long half-life of DRSP combined with a shorter pill-free interval of 4 days. Cycle control was excellent with all three EE/DRSP combinations showing a high level of scheduled bleeding, and a low level of unscheduled bleeding/spotting versus DRSP alone, as could be expected (134). This good control of the cycle reflected a strong endometrial inhibition. In terms of metabolic impact, lipid metabolism showed a slight estrogenic dominance translated into slight, non-statistically significant increases in HDL-C, LDL-C (which is somewhat unusual in relation with estrogen dominance), and total cholesterol, while triglyceride levels increased by 74% vs baseline, an increase which was within the normal range (199). Glucose metabolism, even after one year of use of EE 30 µg/DRSP 3 mg, did not show any clinically meaningful changes (200, 201). Concerning body weight, there was essentially no change in EE/DRSP users. Some studies indicate a slight decrease during the first months of use, with a return to initial weight at one year of treatment, whereas there is frequently a one kg weight gain at one year with other COCs (200, 202).

As relates to blood pressure, it is important to realize that EE strongly activates sodium and water reabsorption because it activates the RAAS system, whereas DRSP, with its 3 mg dosage regimen and its anti-mineralocorticoid effect (corresponding to about 25 mg spironolactone), strongly opposes the RAAS, and is natriuretic, and potassium-sparing. This effective inhibition by DRSP is dose-dependent, as measured by the counter-regulatory increased levels of plasma renin activity and aldosterone, while the aldosterone receptor is blocked by DRSP. A reduction of arteriolar vasoconstriction and excess sympathetic nervous system action, which are also aldosterone effects, are additionally attributable to DRSP (203). It is noticeable that EE/DRSP use does not alter total and extracellular body water and does not change fat mass and fat free mass (204, 205). This good balance between EE and DRSP helps in maintaining blood pressure (BP) at its basal normal level in healthy users, despite most COCs, that lead to a slight elevation of BP (206). Moreover, studies in postmenopausal hypertension-treated women using E2–1 mg/DRSP 3 mg described a significant BP reduction by -8.6 mmHg (SBP)/-5.8 mmHg (DBP) (207).

In terms of tolerability and satisfaction, EE 30 µg/DRSP 3 mg compared to EE 30 µg/LNG 150 µg showed similar beneficial effects on cycle control, water retention and typical adverse events, but EE/DRSP was significantly better in alleviating premenstrual tension, and androgenic symptoms such as acne (decrease by 55%) and hirsutism. It was also superior to EE/LNG in improving physical and emotional well-being (208). Moreover, studies of premenstrual dysphoric disease (PMDD) showed that EE/DRSP 24/4 significantly improved the DRSP score bound to that syndrome (6).

As EE/DRSP is well tolerated, does not alter metabolism, regularizes the menstrual cycle, decreases androgens and androgenic symptoms, does not increase weight, and does not impair blood pressure, this COC is often chosen for treatment of PCOS (anovulation/oligomenorrhea, hirsutism/acne, hyperandrogenism, ovarian cysts, and frequent excess weight). Indeed, EE/DRSP treatment is effective and more anti-androgenic than EE/DNG (5), although less anti-androgenic than EE/CPA (209). These anti-androgenic COCs, although effective for treating the endocrine disorder, are less efficient for mitigating the metabolic disorder of PCOS, which is very near to a Metabolic Syndrome (MetS). This syndrome must include diagnosis of at least 3 findings among the following criteria: hypertension - hyperglycemia - hypertriglyceridemia - low HDL-C - excess fat mass and obesity. Most of these metabolic alterations are present in PCOS women, particularly in those with excess weight. This requires treatment by metformin or, more recently, by a glucagon -like peptide-1 (GLP-1) agonist, in addition to a COC (210–212). Moreover, PCOS has now been recognized as a “risk-enhancing” factor for CVD, including coronary heart disease and stroke (213). PCOS is also associated with increased thrombin generation - independent of other risk factors of CVD - which may add to the CV risks associated with PCOS (164). As administration of a COC is a “first line” beneficial treatment for PCOS (160), then, is EE/DRSP a safe treatment, when we know that PCOS increases cardiovascular risk, including thrombotic risk? Indeed, analysis of individual factors of coagulation and anti-coagulation in users of EE/DRSP (and of all EE-containing COCs) show rather non-significant changes, unable to lead to a correct evaluation of the thrombotic risk (166, 214). However, it is known that synergistic effects of these parameters tend to increase the total thrombogenicity of these COCs (120). Moreover, markers of thrombogenicity such as thrombin generation assessment (TGA) and endogenous thrombin potential – based activated protein C resistance (ETP based – APCr) are significantly increased in EE-COCs users, and particularly with EE/DRSP (215, 216). Additionally, these specific risk markers are in full agreement with large, consensual, meta-analyses of epidemiologic studies showing an increased risk of VTE and ATE with use of EE-COCs [e.g (217) (118).,; and others]. In these studies, EE-LNG shows the lowest risk of VTE (about 2.5-3.0 times the risk of non-users), and EE/DRSP about 1.5 – 2.0 times the adjusted risk of EE-LNG, i.e., one of the highest risks of VTE associated with COCs (105, 118, 217). Moreover, EE/DRSP was compared to another COC-containing EE and the androgenic progestogen NETA in a disproportionality analysis for the risk of VTE using data derived from the FDA Adverse Event Reporting System (FAERS). Here also, the risk was significantly greater for EE/DRSP, with a reporting odds ratio (ROR) of 1.33 to 2.16 according to age vs EE/NETA (218). In contrast, COCs containing natural estrogens (E2, E2V) show, in place of EE, a definitely lower risk of VTE than EE-COCs (106) and, in that PCOS context, would certainly be a safer choice than EE/DRSP, but this is still waiting for a clinical demonstration in the case of E4/DRSP, the third COC containing a natural estrogen (E4).The recent combination of DRSP + E4 contains E4–15 mg as monohydrate + DRSP 3 mg, 24/4 days regimen, and was introduced to the market in 2021. It is the only COC containing E4, and shows some important differences compared to E2 and EE. This natural estrogen, E4, originating from the fetal liver and placenta, is now partly synthesized from plants. Its bioavailability (**Table 5**) is high and its half-life is long compared with EE and E2.

**Table 5:** Pharmacokinetic and metabolic properties of E4, EE, and E2 when used in a combined oral contraceptive. Adapted from (219).

Properties	Estetrol (E4)	Ethinylestradiol (EE)	Estradiol (E2)
Oral bioavailability	70%	40-45%	0.1-12%
Half-life	24 – 28h	5 – 30h	13 – 20h
Free active fraction	50%	1% - 2%	1% - 2%
CYP3A4 metabolism^*^	No	Yes	Yes
Active metabolites	No	Yes^**^	Yes^**^

^*^CYP3A4, cytochrome P450 3A4.

^**^Metabolites that can react and damage the DNA.

E4 metabolism is quite different from the metabolism of E2, as it is insensitive to CYP metabolizing enzymes and has no active metabolites, whereas E2, by contrast, is extensively transformed to many metabolites, some of which are active and may increase thrombin generation, and may in that way explain an increased thrombotic risk attributed partly to oral E2 (220). Moreover, catechol estrogen quinones, metabolized from EE and E2, could serve as initiators of breast and other cancers, which is not the case of E4 (167, 221). E4 acts through activation of the nuclear ER alpha and partly antagonizes the membrane ER alpha, in contrast to EE and E2 that act on both isoforms. E4 shows a selective activity in tissues [Native Estrogen with Selective activity in Tissues (NEST)] with, among others, a low impact on the liver (e.g., low synthesis of binding globulins such as SHBG), low synthesis of angiotensinogen and thereby low activation of the RAAS, low production of hemostasis factors, and low impact on lipid/glucose metabolism (222–224). However, E4 retains major effects of E2 and EE with a lower level of potency, considering that it must be used for contraception at a 15 mg daily dose, instead of 10-30 µg for EE and 1–3 mg for E2. Accordingly, E4–15 mg (equivalent to anhydrate 14.2 mg) is combined with DRSP 3mg in a monophasic 24/4-day regimen, which deeply inhibits gonadotropin secretion and ovarian function (225, 226). This combination has a high level of contraceptive effectiveness, and in two large phase III studies the typical Pearl Index was 0.44 in Europe/Russia and 2.65 in USA/Canada (16, 227). Cycle control with this combination, although containing a natural estrogen, is fine, near to that of EE/DRSP, i.e., with a high level of scheduled bleeding and a low level of unscheduled bleeding (134). In terms of tolerability and safety and essentially based on pooled results of both phase III studies, its tolerance was excellent; metrorrhagia (9.5% of participants), breast pain (4.0%), acne (3.3%), headache (3.2%) were the most frequent adverse events reported (228). The overall discontinuation rate was 21.6%, lowest with E4/DRSP compared to both other estrogen-containing COCs, E2V/DNG and E2/NOMAC (35% and 28%, respectively), and the discontinuation for abnormal bleeding was only 3.4% in E4/DRSP users (134).

As discussed earlier, among risk factors of cardiovascular disorders, increased blood pressure was not observed in E4/DRSP studied users (probably partly due both to E4 induction of a low level of angiotensinogen (224) and to the anti-mineralocorticoid effect of DRSP, reducing RAAS activation) (203), with no clinically significant change of lipids and glucose metabolism, no influence of increased BMI, and no problem of DRSP-associated hyperkalemia (0.2% of participants, who were asymptomatic). In the pooled Phase III studies, 3417 participants completed 35,093 E4/DRSP cycles and only one lower limb VTE was reported at four months of use of E4/DRSP. The overall estimated annual VTE incidence rate of all users was 3.66/10,000 woman-years, whereas the VTE incidence for EE-containing COC users ranges from 5 to 10/10,000 woman-years (228, 229). This very low incidence of VTE has of course to be confirmed by a large and long comparative study of E4/DRSP *vs* other COCs – and *vs* EE, as well as E2-based COCs (PASS study ongoing). Nevertheless, many observations point to a potentially low incidence of VTE with use of E4/DRSP: [1] Hemostasis parameter changes during use of this COC are less or similar to the ones observed with EE/LNG and EE/DRSP (215). [2] In particular, low increase in ETP-based nAPCsr with E4/DRSP (+ 30% vs baseline), whereas higher increases of + 165% for EE/LNG and + 219% for EE/DRSP were recorded (215). [3] Lower thrombin generation assessment was observed in users of E4/DRSP vs EE-containing COCs, indicating a lower risk of VTE for E4/DRSP (165). [4] APCr based on endogenous thrombin potential was proven to be a surrogate marker of VTE as determined in clinical studies (216) and showed that ETP-based nAPCsr value for E4/DRSP was lower than with use of EE-COCs (18, 230). [5] A meta-analysis of E2V/DNG and E2/NOMAC shows that they have a lower VTE risk than EE-COCs (106). [6] A disproportionality analysis of the Eudravigilance database showed a lower reporting rate for VTE risk when comparing E2-based COCs to EE-COCs. Specifically, E4/DRSP showed the lowest proportionality reporting rate (0.12), like the rate for DRSP only, and slightly lower than for E2/NOMAC and E2V/DNG, and largely lower than for all EE-COCs, which show a well-known increased thrombotic risk (107). All these concordant investigations point to the probability of VTE risk in E4/DRSP users to be predicted as very low and once again indicate that estrogen E2, E2V, E4-containing COCs are safer than EE-COCs in terms of thrombotic risk.

As far as ATEs are concerned, DRSP alone was introduced too recently to provide analyzable cohorts of users and for defining a potential risk of ischemic stroke and myocardial infarction. In contrast, EE/DRSP shows in two large Danish studies, slight though significant increases about 1.5 to 2.2 in users vs non-users for both ischemic stroke and myocardial infarction, with an incidence of these events slightly lower with the EE 20 µg than with the EE 30 µg preparation (93, 108). However, in the Yonis study, the MI risk was very low for the EE 20 µg formulation and not reported. Although there is no consensus, Dinger, in a long-term study (mean duration 5.4 years) showed a significant low risk of both ischemic stroke and myocardial infarction in EE/DRSP users compared with users of OCs containing other progestogens. He attributed this observation partly to a potential protective effect of DRSP thanks to its anti-mineralocorticoid effect (231).

In conclusion, due to its anti-mineralocorticoid activity and potent progestogenic and anti-androgenic action, when used alone as a POP, DRSP shows a high contraceptive efficacy, metabolic and clinical tolerance, and opposes water retention, without increasing thrombotic risk. However, it is hampered by suboptimal cycle control, which is fully improved when DRSP is combined with EE, at the expense of an increased thrombotic risk (typical of so-called 3^rd^ and 4^th^ generation COCs). By contrast, when DRSP is combined with natural E4, efficacy, tolerance and control of the cycle are adequate with a predicted lower thrombotic risk, which has still to be clinically demonstrated (studies ongoing) (219, 232).

## 5 Pharmacodynamics summary and conclusions: a shift in COC safety

The introduction of the combined estrogen-progestogen pill to the market in 1960 (Enovid^®^) offered to women not only a new type of contraception, which shows a high effectiveness, but also the possibility of mastering their own fertility and sexuality, a significant social revolution (233). Sixty-five years later, COCs used worldwide still associate an estrogen (mostly EE) with different types of progestogens from highly androgenic to highly anti-androgenic activity. These progressive changes in formulation and lower dosages, particularly in estrogen, were conducted for two main reasons beyond contraceptive efficacy: tolerance and safety.

*Tolerance* responds particularly to women’s needs, extending from better control of the cycle to improvement or disappearance of mastalgia, dysmenorrhea, premenstrual syndrome, with biological, metabolic, bodily, sexual and psychic preservation (7). Provided EE is maintained at a low dose (about 20 µg per pill per day, combined with a progestogen, ideally mildly anti-androgenic, and titrated primarily to achieve endometrial stability), COCs do allow reaching a high level of tolerability (208, 234, 235).

In terms of safety it soon appeared that decreasing the EE dose (234), even to about 10 µg/day (104), reduced the risk of VTE and ATE, though not completely. Further reduction in acquired risk could be obtained rather markedly with the early androgenic progestogens NET, LNG and NGM and somewhat less androgenic DSG and GSD. They had to be combined with adequate doses of EE to minimize cyclic disorders, atherogenic lipid shift to more LDL-C and less HDL-C, hyperinsulinism, decreases in binding proteins such as SHBG, trend to weight increase and skin/hair problems, which are more important according to that androgenic strength of progestogens. All these parameters were also optimized with newer anti-androgenic progestogens CPA, CMA, DNG, NOMAC and DRSP, either combined with EE, or with natural estrogens for the last 3 of them (73, 119). Among these progestogens, DRSP is the only one to show a clinically significant anti-aldosterone effect with anti-hypertensive action (198, 203, 236). Moreover, use of POPs is well tolerated, except for irregular bleeding, a major adverse event that can lead to lack of adherence, and to discontinuation of treatment.

*Safety* also requires consideration of different important aspects of risk of cancer, meningiomas and, specifically, thrombotic risk in users of COCs and POPs.

These hormonal contraceptives are known to be involved in cancer risks, which are largely detailed in other publications. Indeed, COCs (including newer products), POPs and also LNG-IUDs decrease the risk of endometrial cancer and ovarian cancer (237–239), while this is less demonstrated for colorectal cancer (237). By contrast, recent studies show an increased risk of breast cancer with current or recent use, in women <50 years of age, of some COCs and POPs, and LNG-IUDs. This increase is small, though significant of the order of 1.2 to 1.3 vs non-users, meaning about one extra breast cancer for every 7690 to 7752 women using a hormonal contraceptive for one year (240–243). In a recently reported nationwide Swedish study (243), the risk of breast cancer is slightly though significantly more elevated in users of progestogens alone (HR 1.21) vs non-users, than with any combined EE-COC (HR 1.12). The authors even suggested that “estrogen may attenuate progestin’s harmful effect”; In terms of risk, it is too early to obtain comparative results between EE and bio-identical estrogens in combined OCs. It is interesting to note that in postmenopausal women using MHT, the estrogen - essentially E2 or CEE - when used alone increases the risk of breast cancer less than its association with synthetic progestogens (244).

In terms of severe adverse - although rare - events, it must be realized that the administration of CPA, CMA, and NOMAC at high dosages for gynecological and sexual disorders (CPA) entail the development of meningiomas (mostly benign), in a dose-dependent manner. Meningiomas could be diagnosed already after 6–12 months of administration of particularly high dosages of these progestogens (e.g. CPA 25–100 mg/day) but more often after some years. However, if long-term use of these progestins at high dose is scheduled, recommendations (including the French Haute Autorité de la Santé [HAS] and others) are to perform magnetic resonance imaging (MRI) at regular intervals, and, if meningiomas are developing, cessation of the progestogen, MRI, and neurological surveillance, which can lead to neurosurgery (245, 246). There is until now no consistent demonstration that COC and POP use, with their low content in those progestogens, do increase the risk of meningiomas (149).

Another important aspect of safety is concerned with the persistent thrombotic risk of VTE and ATE associated with COC use, while, as carefully reviewed (92, 247) POPs do not increase VTE or ATE, or very slightly (93), allowing their safe prescription in case of estrogen contraindication, including thrombotic risk. Indeed, the risk of COCs is mainly attributed to the estrogen component, historically and essentially EE, modulated by the accompanying progestogen. In Europe, about 22,000 VTE cases related to COC use occur each year and the estimated risk to develop VTE in users is, according to EMA, of 5-7/10,000 women-years (WY) vs 2/10,000 WY in non-pregnant non-users (138, 248). Understandably, this major risk was calculated for the different COCs progressively entering the market, to determine if there were differences in risk according to formulation. Indeed, hemostatic studies have shown that under increased hepatic synthesis of proteins, including hemostasis factors, due to EE, some small increases in individual procoagulant factors and decreases in anti-coagulants were observed - a profile of coagulation not clearly able to lead to thrombosis (166). However, more global studies of thrombin generation and of resistance to the activated protein C indicated with more evidence that the COC users’ hemostatic profile may correspond to an increased thrombotic risk (165, 215, 249, 250). In this situation, larger and longer epidemiological studies of occurrence of VTE allowed to establish a rather consensual hierarchy of thrombotic risk in EE-containing COCs. Those containing EE and a strongly androgenic progestin (NET, or LNG, or NGM) yielded a “low” level of VTE risk while EE combined with a mildly androgenic progestin (DSG or GSD) showed a significantly increased level of risk compared with the latter. Moreover, COCs containing EE and an anti-androgenic progestogen (i.e. EE/DRSP, CMA, CPA and DNG) also showed a similar level of risk of VTE as EE/DSG or GSD (104). Consequently, agencies have established that EE (<50 µg, usually 30 or 20 µg/day) combined with LNG shows the lowest thrombotic risk in COC users and is considered until now (for about the last 12 years) as the safest combined oral contraceptive (138). However, this combination is still responsible for a significant number of vascular events and is also accompanied by tolerance problems (due mainly to the strong androgenic effects of LNG).

As potent EE remains the principal cause of hepatic hemostasis effects, combinations containing natural estrogens such as E2V/DNG, E2/NOMAC and E4/DRSP have been introduced to the market in 2009, 2011, and 2021, respectively. Although E2V is a synthetic estrogen, it is readily converted to E2 during the first hepatic pass. Natural estrogens have a much lower clinical and biological impact than EE, including the coagulation system (137, 167). All three natural estrogen-containing COCs have a high level of contraceptive efficacy, nearly neutral biologic and metabolic impact and good tolerance. Concerning cycle control, they all entail a low and appropriate level of unscheduled bleeding, while E2- and E2V-COCs show a reduced level of scheduled bleeding (~ 70%) *vs* ~ 85-90% for E4/DRSP, like EE-COCs (134). Studies of individual hemostasis factors in users of natural estrogen COCs showed only small changes, and, more important, global assays which can indicate a potential thrombotic risk (thrombin generation, and ETP-based-nAPCsr assessments) showed a lower risk profile than EE-COCs (230). Moreover, a pooled study of E2V/DNG showed a significantly decreased VTE risk *vs* EE/LNG (142), while a meta-analysis of both E2V/DNG and E2/NOMAC showed a significant 33% reduction in VTE risk *vs* EE-COCs (106). Finally, a disproportionality analysis of the Eudravigilance database supports the safer thrombotic profile of natural estrogen-COCs over EE-COCs (107). Similar to E2V/DNG and E2/NOMAC earlier trials, an E4/DRSP PASS study for clinical demonstration of a potential low level of thrombotic risk is now ongoing.

Much evidence is emerging that natural estrogen-based COCs - or one of them - may supplant LNG-COCs (and other EE-COCs) as a paradigm of vascular safety that is long waited for and may respond even better to women’s contraceptive needs, particularly in the safety domain.

Would it be time for the EMA to shift its paradigm of vascular safety from EE/LNG to a natural estrogen-COC? (106, 232).
