# Supplementary material for: A detailed review of pharmacokinetics/pharmacodynamics of progestogens in oral contraception
Source: Front Endocrinol (Lausanne). 2026 Mar 26;17:1730768. doi: 10.3389/fendo.2026.1730768 (PMC13061722; doi:10.3389/fendo.2026.1730768)
Supplement: Supplementary file 1 [file Table1.docx]

Supplementary Material

Supplementary Table: Concentrations, Tmax and area under the curve (AUC) of progestogens when administered orally combined with estrogens after multiple dose administration

|  | Combined estrogen | Cmax  (ng/mL) | Tmax  (h) | AUC  (ng.h/mL) | References |
| --- | --- | --- | --- | --- | --- |
| NET 1 mg | EE | 23.5 | 1.3 | 167 | (251) |
| LNG 0.150mg | EE | 5.0-7.0 | 1.5-2.0 | 20.4-85.8 | (25,27,252) |
| NGM 0.250mg  (active NGMN metabolite) | EE | 1.8-2.19 | 1.0-1.5 | 16.8-19.9 | (34,35,37,253) |
| DSG 0.150mg  (active ETN metabolite) | EE | 3.69-6.36 | 1.6-1.75 | 32.0-45.5 | (39,254,255) |
| GSD 0.075mg | EE | 12-19 | 0.4-0.9 | 135-242 | (44,256,257) |
| DNG 3mg | E2V | 82.9 | 1.5 | 809 | (258) |
| CMA 2mg | EE | 2.01-2.21 | 1.40-1.61 | 15.8-19.4 | (52) |
| CPA 2mg | EE | 21.0-23.9 | 1.4 | 169-183 | (55) |
| NOMAC 2.5mg | E2 | 12.3 | 1.5 | 106 | (57) |
| DRSP 3mg | EE | 60-87 | 1-2 | 763 | (60,61) |
